# Identifying metabolic pathways for production of extracellular polymeric substances by the diatom *Fragilariopsis cylindrus* inhabiting sea ice

**DOI:** 10.1038/s41396-017-0039-z

**Published:** 2018-01-18

**Authors:** Shazia N. Aslam, Jan Strauss, David N. Thomas, Thomas Mock, Graham J. C. Underwood

**Affiliations:** 1 School of Biological Sciences, University of Essex, Wivenhoe Park, Colchester, CO4 3SQ UK; 2 University of East Anglia, School of Environmental Sciences, Norwich Park, Norwich, NR4 7TJ UK; 3 European Molecular Biology Laboratory (EMBL) Hamburg, c/o German Electron Synchrotron (DESY), Notkestraße 85, 22607 Hamburg, Germany; 4 School of Ocean Sciences, College of Natural Science, Bangor University, Menai Bridge, Anglesey, LL59 5AB UK; 5 Marine Research Centre, Finnish Environment Institute (SYKE), Helsinki, Finland

## Abstract

Diatoms are significant primary producers in sea ice, an ephemeral habitat with steep vertical gradients of temperature and salinity characterizing the ice matrix environment. To cope with the variable and challenging conditions, sea ice diatoms produce polysaccharide-rich extracellular polymeric substances (EPS) that play important roles in adhesion, cell protection, ligand binding and as organic carbon sources. Significant differences in EPS concentrations and chemical composition corresponding to temperature and salinity gradients were present in sea ice from the Weddell Sea and Eastern Antarctic regions of the Southern Ocean. To reconstruct the first metabolic pathway for EPS production in diatoms, we exposed *Fragilariopsis cylindrus*, a key bi-polar diatom species, to simulated sea ice formation. Transcriptome profiling under varying conditions of EPS production identified a significant number of genes and divergent alleles. Their complex differential expression patterns under simulated sea ice formation was aligned with physiological and biochemical properties of the cells, and with field measurements of sea ice EPS characteristics. Thus, the molecular complexity of the EPS pathway suggests metabolic plasticity in *F. cylindrus* is required to cope with the challenging conditions of the highly variable and extreme sea ice habitat.

## Introduction

The production of polysaccharide-rich extracellular polymeric substances (EPS) by microorganisms is ubiquitous in many environments: water, soils, benign and pathogenic biofilms [1, 2], where EPS play important roles in cell adhesion, cell signalling, ligand binding and as a carbon source [3–5]. The production of EPS is a characteristic of many diatoms [6]; an algal group which contributes 20% of annual global carbon fixation [7]. EPS production is particularly a feature of the pennate diatoms that are dominant in autotrophic biofilms [8, 9] and in sea ice microbial assemblages [10].

In sea ice, a biome covering up to 15% of the world’s ocean area and which supports productive microbial communities within the semi-solid ice-water matrix [11–13], 40% of the dissolved organic matter (DOM) present is EPS produced by diatoms [14–16]. EPS and DOM modify the physical structure of the ice-water matrix [17, 18], provide a rich carbohydrate source [19], and on ice melt, contribute to the stimulation of water-column carbon cycling [13, 20, 21], influencing vertical carbon fluxes to deeper polar waters [22, 23] and the production of atmospherically active polar aerosols [24].

Despite the importance of diatom productivity in EPS sea ice carbon biogeochemistry [25], together with current changes in the distribution and thickness of polar sea ice [26], little is known about the expression of genes involved in the synthesis and excretion of EPS in diatoms [1, 27] and the biosynthesis pathway of complex EPS is completely unknown [28]. The main pathways of photosynthesis and carbohydrate metabolism in diatoms have been reconstructed [27, 29, 30]. Polysaccharide production pathways are conserved across the prokaryotes and eukaryotes [1, 31], with monosaccharides converted to nucleotide sugars and assembled into polysaccharides by the action of glycosyltransferases (GTs) [3, 27]. In diatoms, EPS are assembled in the Golgi apparatus and transported in vesicles to the cell membrane and excreted [32]. These EPS then undergo further self-assembly in the external environment and form cell frustule coatings, adhesive structures or are used in locomotion [32–34]. Diatoms change the rates of production and chemistry of their EPS in response to external factors such as nutrients, light and salinity stress [35–37], but there are no studies on how the pathways underlying EPS production respond to the environmental drivers that shape the ecological role of this successful algal group.

**Fig. 1:**
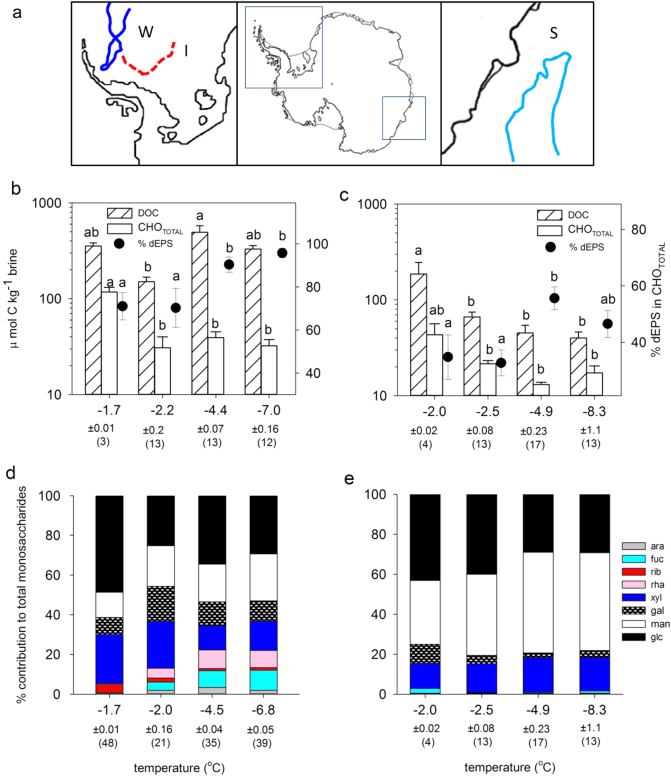
**a** Cruise tracks in the Weddell Sea (from WWOS (W) and ISPOL (I) cruises) and off East Antarctica (SIPEX (S)). **b** Dissolved organic carbon (DOC), total carbohydrate (CHO_TOTAL_) concentrations and the % contribution of dissolved EPS (dEPS) in sea ice brine samples from the Weddell Sea and **c** in sea ice core samples from East Antarctica (means ± standard error, significant differences (*p* < 0.05 or less) between variables indicated by different letter codes); grouped by temperature categories (standard error ± <0.16 °C), with the corresponding % relative abundance of the monosaccharide composition in dEPS in **d** Weddell Sea brines and **e** SIPEX sea ice cores. Variation in temperature values and number of replicates between **b** and **d** due to subsampling of EPS fractions and sample losses

We investigated the characteristics of EPS from ice cores and ice brines from sea ice present in the Weddell Sea and the East Antarctic ocean sampled across winter–spring–summer transitions, and exposed to a range of temperature and salinity conditions (Fig. 1). Based on these field observations, we designed a laboratory investigation to allow the first reconstruction of a metabolic pathway of EPS production linked to physiological measurements in a polar diatom, as a model for the major environmental transitions experienced by the majority of microorganisms responsible for EPS production in polar oceans. *Fragilariopsis cylindrus* is widely distributed in the Arctic and Southern Oceans associated with sea ice [10, 38–40]. Its genome sequence is the first to be completed for any eukaryotic psychrophilic organism, and has revealed key evolutionary adaptations to living in polar oceans [41], with a distinct phenotypic plasticity [40–42], able to grow at −4 °C, with growth rate decreasing significantly at −8 °C [36, 43]. It produces a range of EPS, whose composition corresponds with that of EPS in natural sea ice [25, 36].

**Fig. 2:**
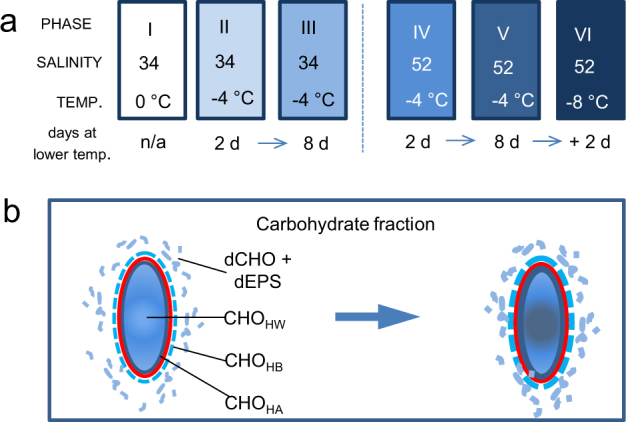
**a** Six experimental phases, with the temperature and salinity conditions used in this study. (I) Temperture of 0 °C and salinity 34 (seawater before ice formation), (II) 2 days at −4 °C and salinity 34 (early freezing or frazil ice formation), (III) 8 days at −4 °C and salinity 34 (late freezing, frazil ice layer formation), (IV) 2 days at −4 °C and salinity 52 (initialization of brine channel formation in ice), (V) 8 days at −4 °C and salinity 52 (established brine channel formation) and (VI) further temperature stress, 8 days at −4 °C followed by 2 days at −8 °C, and salinity 52. **b** Four diatom-associated carbohydrate fractions investigated; (dCHO+dEPS), extracellular dissolved carbohydrate and dissolved extracellular polymeric substances; (CHO_HW_), intracellular carbohydrate, isolated in hot water extracts; (CHO_HB_), extracellular tightly bound carbohydrates/EPS, solubilised in a hot bicarbonate extraction; (CHO_HA_), carbohydrate liberated from dissolving silica frustules in hot alkali; and the trend of increasing carbohydrate yields across the six experimental phases

*Fragilariopsis cylindrus* was grown across a matrix of salinity and temperature conditions, from open-water to sea ice brines of salinity 52 and temperatures of −8 °C (Fig. 2), measuring cell physiology, yields and chemical composition of EPS, and the expression patterns of key genes involved in carbohydrate synthesis and polysaccharide production. We reconstructed the first putative metabolic pathway for EPS production in diatoms, and showed three different responses of this pathway in relation to salinity and temperature. These results concur with field measurements of sea ice EPS, and provide new insights into the physiological flexibility of diatoms, and helps to explain their success in one of the most extreme and globally significant biomes.

## Materials and methods

### Field sample collection and determination of EPS in Antarctic sea ice

Samples (sea ice brines, ice cores) were collected during two cruises to the Weddell Sea, Antarctica, in December 2004 (ISPOL) and September–October 2006 (WWOS) [16, 44], and during the Sea Ice Physics and Ecosystems Experiment (SIPEX) research expedition to Eastern Antarctica (110°–130°E, between September and October 2007 [45], thus encompassing contrasting sea ice conditions and types from winter to summer in the Weddell Sea, and from a winter to spring transition (SIPEX) (Fig. 1a). All measurements described here were obtained from opportunistically collected samples (see [44] for details) from sea ice brines collected with the sackhole sampling technique, and bulk sea ice from melted 10-cm-thick ice core segments (see [16]). Samples were filtered through pre-combusted GF/F filters (Whatman, 0.7 µm) and filters and filtrates were stored at −20 °C until further analysis.

Brine and ice core samples were analysed for dissolved organic carbon (DOC), total (CHO_TOTAL_) and dissolved carbohydrates (dCHOs) and dissolved EPS (dEPS) concentrations, and EPS monosaccharide composition [25]. Data for the Weddell and East Antarctic regions were compared across four temperature subsets; bottom ice at the seawater interface, with temperatures of −1.7 °C; and ice cores or brines in decreasing temperature bands of approximately −2 °C, −4 °C and −8 °C (Fig. 1b, c).

### Cell culture experimental conditions

Axenic cultures of *F. cylindrus* (CCMP1102) were grown in enriched artificial seawater media [36]. Separate stock cultures were acclimated at two salinities (34 and 52) over a 3-month period prior to the experiment to avoid the negative but transient impacts of acute changes in salinity on diatom photophysiology [36, 43, 46]. These acclimated cells were then used to establish a set of triplicate control and temperature-reduction cultures (see Supplementary Information).

A stepwise reduction in temperatures was used (with a partially repeated measures sampling design, see Supplementary Information) to follow the response of cells to a range of temperature and salinity conditions representing phases in the development of sea ice (cf. [47]): phase I, normal growth conditions in seawater (34 salinity and 0 °C) before ice formation; phase II, early freezing or frazil ice formation (salinity 34, 2 days exposure to −4 °C); phase III, continuing cold condition (salinity 34, 8 days exposure to −4 °C); phase IV, initiation of brine channel formation and trapping of cells in pancake ice (salinity 52, 2 days exposure to −4 °C); phase V was a continuation of these conditions (8 days exposure to −4 °C) and Phase VI simulated a further temperature stress (2 days exposure to −8 °C after 8 days exposure to −4 °C under phase V), as found in colder sea ice brine channels while maintaining salinity at 52 (Fig. 2a).

Cells (initial density of 1 × 10^5^ cells mL^−1^) were grown in plastic bottles (5 L, containing 3 L of media). Treatments and controls (maintained at 0 °C throughout) were established in triplicate for both salinity 34 and 52 conditions. Triplicates were grown for 12 days at 0 °C, with a 50% volume media change at day 10 (to reduce any potential for nutrient limitation [36] see Supplementary Information), before the temperature reductions commenced. Designated flasks were reduced to −4 °C on day 12, and first measured on day 14 (phases I, II and IV), on day 20 (phase III and V) and on day 22 after a further decrease to −8 °C for 2 days (phase VI). Subsamples were taken for measurements of cell density, cell photophysiology, carbohydrate content, biochemical composition and RNA extraction. Intrinsic growth rate (µ per day) and Chlorophyll *a* concentration was determined at each time point [36].

### Cell photophysiology, carbohydrate and EPS production and composition

Cell maximum PSII photochemical efficiency (*F*_v_/*F*_m_) and functional absorbance cross-section of photosystem II (*σ*_PSII_, nm^2^ per RCII) was determined using a Satlantic FIRe fluorometer (Satlantic Inc. Halifax, Canada) [36, 48]. Carbohydrates (Fig. 2b) were fractionated into dCHO containing dEPS (precipitation with 30 and 70% ethanol, termed dEPS_complex_ and dEPS, respectively, [15]) and non-polymeric lower molecular weight carbohydrates secreted by cells, and particulate carbohydrates, by filtration [36]. Pellets of cells and associated particulate carbohydrates were sequentially extracted [36] to obtain a hot water-extracted carbohydrate (CHO_HW_) fraction (mainly intracellular storage polysaccharides), a hot bicarbonate-extracted (CHO_HB_) fraction (solubilising gelatinous and water-insoluble EPS such as pads and gels) and a hot alkali extraction (CHO_HA_) liberating EPS associated with the silica frustules [25]. Carbohydrate concentrations in each fraction were determined using a modified phenol sulphuric acid assay, uronic acids were determined by standard carbazole assay and neutral monosaccharide composition was determined by gas chromatography linked with mass spectroscopy [16, 36].

### RNA extraction, RNA-seq library preparation and sequencing

Cells were filtered onto Isopore Polycarbonate filters (1.2 µm, 47 mm, Merck Millipore, Darmstadt, Germany), immediately frozen in liquid nitrogen and stored at −80 °C. In phase III, V and VI, the media froze in one of the three flasks, and we did not extract RNA from that replicate, resulting in *n* = 2. Total RNA was extracted using a TRIzol protocol [49]. Preparation of 50 bp paired-end libraries and RNA-sequencing with a HiSeq2000 instrument (Illumina, San Diego, CA, USA) was performed at the Earlham Institute (Norwich, UK). After initial RNA quality checks, multiplexed cDNA libraries were constructed, with each library pool run in a single lane. Sequencing reads were de-multiplexed using CASAVA (Illumina, San Diego, CA, USA), allowing for a one base-pair mismatch per library. Sequencing data was cleaned using Trim Galore! v0.4.4 [50] with FastQC v0.11.5 [51] and Cutadapt v1.14 [52]. Results were summarized in a single report using MultiQC v1.2 [53]. The RNA-sequencing (RNA-seq) aligner STAR v2.5.3a [54] was used to align reads to the *F*. *cylindrus* genome assembly v1.0 (Fracy1_assembly_scaffolds.fasta.gz; http://genome.jgi-psf.org/Fracy1/) allowing for a maximum of two mismatches ${(--{\tt outFilterMismatchNmax 2})}$ to ensure stringent alignment of reads to divergent alleles and allele-specific RNA-seq analysis [41]. The programme featureCounts [55] implemented in the Bioconductor R subread package was used to count reads.

### Differential expression and gene ontology enrichment analysis

Differential gene expression and multidimensional scaling (MDS) analysis was performed using edgeR [56]. To detect differentially expressed genes, pair-wise multiple comparisons were performed between experimental treatments using the glm likelihood ratio test [57] and *p* values were corrected for multiple testing [58]. A functional gene ontology (GO) analysis was performed on differentially expressed genes (*p* < 0.05) using goseq [59] with default statistical testing methods [60]. The GO term annotations associated with each gene and gene length were extracted from the *F*. *cylindrus* genome annotation file using customized Perl scripts. Enriched GO terms (*p* < 0.05) were summarized and visualized using REVIGO [61].

### Identification and hierarchical clustering analysis of carbohydrate-related proteins

Genes encoding for carbohydrate-active enzymes (CAZy) were identified based on homology with biochemically characterized proteins from the CAZy database (www.cazy.org, [62]) to perform a hierarchical clustering analysis of associated mean fragments per kilobase of transcript per million mapped reads (FPKM) expression values. A one minus Pearson's correlation distance metric and the average linking method was applied to cluster genes and results visualised using the Bioconductor R ComplexHeatmap package [63].

### Reconstruction of a hypothetical EPS pathway map

A draft reconstruction of carbohydrate metabolic pathways leading to EPS production was generated based on the annotated set of carbohydrate-active enzymes and manual curation of metabolic genes from the most recent annotation of the *F*. *cylindrus* genome (http://genome.jgi.doe.gov/Fracy1/) using GO, KEGG pathways and clusters of eukaryotic orthologous groups (KOG) of proteins [64] information. Additionally, using canonical polysaccharide biosynthesis pathways [29, 65] and bibliographic resources for phylogenetically close organisms [1, 29–31, 33], metabolic genes were collected using BLAST [66] searches, and targeted searches for EC numbers [39] and keywords. For the reconstruction of the pathway map, we analysed all collected metabolic genes for the presence of signal peptides using SignalP [67], selecting only proteins that are predicted to be cytosolic, endoplasmic reticulum (ER) or Golgi enzymes and lack any conserved plastid (ASAF) or mitochondrial targeting sequences. We partially cross-checked identified GTs for conserved ER and Golgi targeting sequences using the database LogSigDB [68], but refrained from an in-depth analysis of ER and Golgi targeting motifs given the lack of specific data for targeting to, and retention of, proteins in the diatom Golgi. The EPS pathway was assembled starting from the canonical polysaccharide pathways, and metabolic reactions catalysed by identified gene products were connected based on EC numbers as informed by KEGG and BRENDA biochemical reaction databases and mapped to the experimental gene expression data during sea ice formation.

### Statistical analysis

Statistical analyses were conducted using SPSS^®^ 18.0 and Minitab v.13.3 (Minitab Inc). Significant differences were determined using *t* test and analysis of variance (ANOVA, with Tukey's post hoc tests). Data were tested for normality and homogeneity of variances (Shapiro–Wilk test, Levene test) and log transformation was done on data deviating from these assumptions. All statistically significant differences quoted are at *p* ≤ 0.05 or less (two-tailed). Monosaccharide compositional data for carbohydrate fractions were analysed using ANOSIM and SIMPER (Primer v.6, Plymouth, UK). Canonical correspondence analysis (CCA) was used to extract the major significant relationships present between the physiological, biochemical and transcriptome datasets using the data values for each treatment (using MVSP v3.1, Kolvec Ltd, North Wales, UK).

### Availability of data and materials

Protocols and full details are given in the Supplementary Information. RNA-seq data are available in the ArrayExpress database (www.ebi.ac.uk/arrayexpress) under accession number E-MTAB-5153.

## Results

### Distribution and chemistry of EPS in Antarctic sea ice

There were significant differences in the concentrations and quality of DOM, CHO_TOTAL_ and dEPS with decreasing temperatures in sea ice brine samples from the Weddell Sea (Fig. 1b, d) and bulk ice samples from Eastern Antarctic Ocean (Fig. 1c, e). High concentrations of DOC and CHO_TOTAL_ were present in samples from the seawater–ice interface (temperatures of −1.7 °C, −2.0 °C for Weddell Sea and East Antarctica, Fig. 1b, c), while CHO_TOTAL_ concentrations decreased in colder brine samples. Similar decreases in concentrations were found in bulk ice concentrations (Fig. 1c). In both brine and bulk ice, the percentage contribution of EPS to CHO_TOTAL_ concentrations increased significantly with decreasing temperatures (from 70 to 95% in brines, and from 35 to 45 to 55% in bulk ice (*p* < 0.01), Fig. 1b, c). Coupled with this increase in % dEPS were significant changes in the monosaccharide composition of the EPS in colder ice brines (with significantly lower relative abundance of glucose (Glc), and increases in galactose (Gal), mannose (Man), fucose (Fuc) and rhamnose (Rha), *p* < 0.001), and similarly in bulk ice samples (decreases in Glc and increases in Man, *p* < 0.05) (Fig. 1d, e).

### Changes in *F. cylindrus* growth and photophysiology

**Fig. 3:**
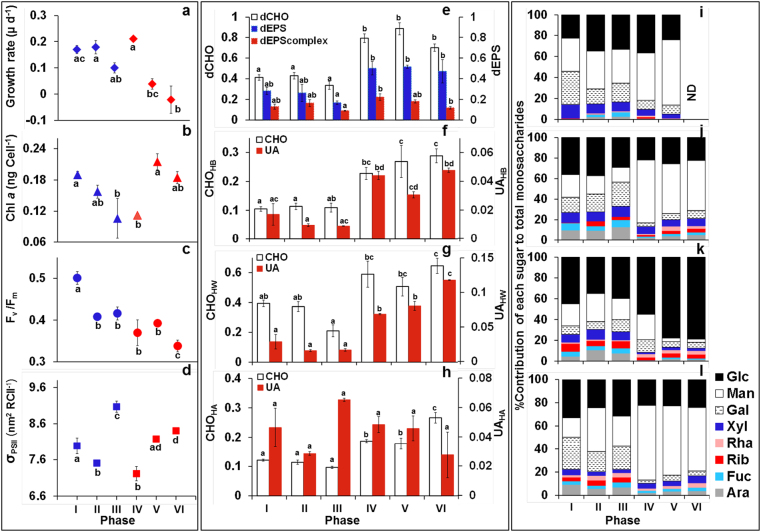
**a** Intrinsic growth rate (µ per day), **b** cell chlorophyll *a* content (ng per cell), **c** maximum PSII photochemical efficiency (*F*_v_/*F*_m_), **d** effective cross-sectional area of PSII (*δ*_PSII_) (mean ± standard error, significant differences between variables in ice stages (*p* < 0.05 or less) indicated by different letter codes); average yield (mean ± standard error) of phenol sulphuric acid quantified carbohydrates pg C per cell) in **e** dissolved carbohydrate (dCHO), including dEPS and dEPS_complex_ components; **f** hot bicarbonate-extracted (CHO_HB_) carbohydrates and uronic acids (UA); **g** hot water-extracted (CHO_HW_) carbohydrates and uronic acids (UA), **h** hot alkali-extracted (CHO_HA_) carbohydrates and uronic acids (UA) fractions, with the corresponding % relative abundance of the monosaccharide composition of **i** dCHO, **j** CHO_HB_, **k** CHO_HW_ and **l** CHO_HA_ extracted fractions from *Fragilariopsis cylindrus* cultures growing under six experimental phases.

*Fragilariopsis cylindrus* grew rapidly (intrinsic growth rate µ per day = 0.17 ± 0.02) at salinity 34 and 0 °C (phase I), with high values of *F*_v_/*F*_m_ and Chl *a* per cell (Fig. 3a–c). Growth rates were not reduced by lowering temperatures (−4 °C after 2 days, phase II) (Fig. 3a). Cells at higher salinity (phase IV, 52 salinity) initially maintained growth rates or Chl *a* per cell compared to phases II or III, but growth rates significantly declined (*p* < 0.01) after 8 days at −4 °C (phase V). Chl *a* per cell increased after prolonged exposure to −4 °C and −8 °C (phases V, VI) (Fig. 3b). Significant declines in *F*_v_/*F*_m_ were associated with slower growth at lower temperatures (Fig. 3c), but were independent of salinity (no significant differences between phases II, III, IV and V), and decreased further at −8 °C (phase VI, Fig. 3c). Decreases in temperature resulted in initial declines in *σ*_PSII_, followed by significant increases in cells exposed to lower temperatures for more than 2 days (phases III, V and VI) (Fig. 3d). *σ*_PSII_ was negatively correlated with growth rate (*r* = −0.578, *n* = 18 at *p* < 0.05).

### Changing yields and chemical composition of carbohydrates

During phase I, *F. cylindrus* produced yields of 0.4 pg C per cell of dCHO, of which 75% was dEPS containing mainly Man, Gal, Glc and xylose (Xyl) (Fig. 3e, i). Yields of dCHO remained unchanged between phases I and III (Fig. 3e), but there were significant changes in the monosaccharide composition of dEPS (analysis of similarities (ANOSIM) global *R* = 0.923, *p* < 0.001) with increased proportions of Man, Fuc and Glc, and reductions in Gal when temperatures reduced to −4 °C (Fig. 3i). The proportion of dEPS (and dEPS_complex_) in dCHO decreased during longer exposure to −4 °C (phases II to III, Fig. 3e) due to increased production of non-EPS carbohydrates in this fraction. Yields of complex extracellular mucilages (CHO_HB_) were lower than those of dCHO, and did not change as temperature decreased to −4 °C (phases I to III, Fig. 3f). The composition of CHO_HB_ changed with lower temperatures (phases II and III), with decreased uronic acid content (from 20 to 10% of CHO_HB_, Fig. 3f), significant decreases in Man and increases in Xyl, arabinose (Ara), Fuc and Gal (global *R* = 0.9, *p* < 0.001) (Fig. 3j).

Higher salinity at −4 °C (phase IV) induced significant increases in yields of dCHO, CHO_HB_ and dEPS (63% of dCHO at phases IV and V, 70% at phase VI), and increased contributions of uronic acids in CHO_HB_ (Fig. 3e, f). Increased salinity resulted in significant declines in Fuc and Gal and increased abundance of Man (*p* < 0.001), resulting in a Glc-Man-rich profile of dCHO and CHO_HB_ (Fig. 3i, j). The Man content of dCHO increased with further exposure to low temperatures (8 days at −4 °C, phase V, Fig. 3i). Exposure to −8 °C for 2 days (phase VI) did not significantly affect yields of dCHO or CHO_HB_ or composition of CHO_HB_ (phase VI composition data for dCHO were lost due to an instrument failure).

Yields of intracellular storage carbohydrates (CHO_HW_) of 0.4 pg C per cell during phases I and II decreased significantly (*p* < 0.01) when cells experienced low temperature (−4 °C) for more than 8 days (phase III, Fig. 3g). The uronic acid content of CHO_HW_ was low, with no change in monosaccharide composition between phases I, II or III (global *R* = 0.333). Increased salinity significantly increased (*p* < 0.05) CHO_HW_ yields (Fig. 3g). Subsequent temperature changes did not affect yield, but extended periods at −4 °C, and 2 days at −8 °C (phases V and VI) resulted in significant declines in Ara, Gal, Xyl and Man content, with CHO_HW_ becoming dominated by Glc (from 40 to 80%, Fig. 3k), suggesting an increased contribution of chrysolaminarin in CHO_HW_.

Frustule-associated carbohydrate (CHO_HA_) showed no significant changes in yield as temperature decreased to −4 °C over 8 days (phases I to III, Fig. 3h). CHO_HA_ had a high uronic acid content (Fig. 3h), and Glc, Gal, Man, Ara and Xyl as the main monosaccharides (Fig. 3l), with decreasing temperature resulting in significant increases in Man (phase I to II). CHO_HA_ yields were significantly higher (*p* < 0.01) at salinity 52 (phases IV to VI), with substantial increases in Man, and declines in other monosaccharides (Fig. 3l). This Man-rich composition was maintained throughout phases V and VI (Fig. 3l), with maximal yields per cell after 2 days at −8 °C (Fig. 3h).

### Identification of genes involved in the production of EPS

**Fig. 4:**
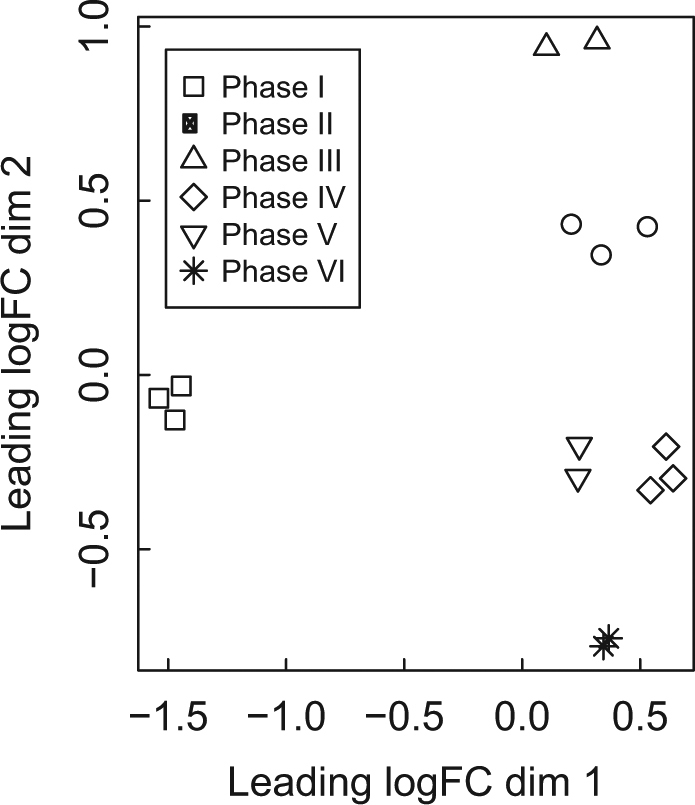
Multidimensional scaling (MDS) plot of digital gene expression profiles for *Fragilariopsis cylindrus* RNA-seq libraries. Distances on the plot reflect the coefficient of variation of expression between samples from a top set of 5000 genes with highest biological variation. Data were normalized according to edgeR’s TMM scaling method

MDS of digital gene expression profiles of *F. cylindrus* revealed a clear separation between open-water conditions (phase I) and all other phases (dimension 1, Fig. 4, Fig. S3). This primary separation (associated with lowered temperature) represented a significant upregulation in transcripts involved in translation (translational initiation and elongation) and RNA metabolic processes (RNA and rRNA processing, pseudouridine synthesis), carbohydrate metabolic processes (gluconeogenesis, glycolytic process), as well as transport and photosynthetic metabolic processes (Fig. S1a–e). Continuing low temperatures (−4 °C, Fig. 2a) caused further reprogramming of the *F. cylindrus* transcriptome, with phases II to VI separating on MDS dimension 2 (Fig. 4). Eight days at −4 °C (phase III) increased the expression of genes involved in RNA and rRNA processing, gluconeogenesis, photosynthesis and light-harvesting and metabolic processes (Fig. S1b). Acclimation to higher salinity (phases IV, V and VI, clustered on MDS dimension 2, Fig. 4) increased the expression of genes involved in glycolytic processes, translational initiation, RNA and rRNA processing, transport and photosynthesis and light-harvesting processes (Fig. S1c,d), and in phase VI, rRNA processing, metabolic processes and photosynthesis and light-harvesting (Fig. S1e). These changes corresponded to major changes in photophysiology, carbohydrate yields and composition (Fig. 3).

We integrated data from carbohydrate biosynthetic pathways (based on EC numbers, KEGG and BRENDA biochemical reaction databases), RNA-seq, including GTs, ATP-binding cassette (ABC) transporters and translocating ATPases, yields and chemical composition of carbohydrates, to identify genes involved in EPS synthesis. Genes encoding for 195 carbohydrate-active enzymes were identified (using CAZy [62]) in the genome of *F. cylindrus* (excluding divergent allelic gene copies, Table S1), including 65 glycoside hydrolases (GHs) and 116 GTs. Expression patterns varied, with three gene expression clusters related to phase: maximal expression in phase I (cluster 1), maximal expression in phase III (cluster 2) and maximal expression in phase VI (cluster 3) (Fig. S2). The largest clusters of strongly induced genes were observed in phases I and VI, with a large cluster of down-regulated genes in phase III (8 days exposure to −4 °C). A number of GHs were up-regulated in phases I, II and III (Table S1).

**Fig. 5:**
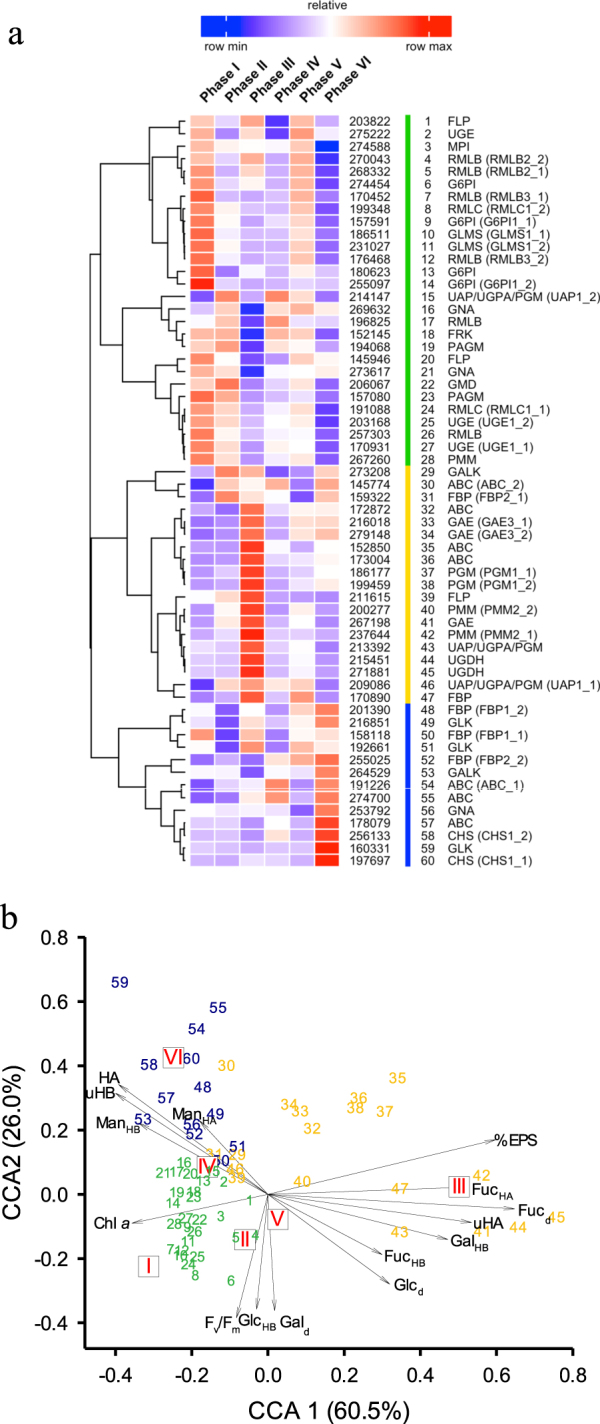
**a** Hierarchical clustering analysis of expression values (mean fragments per kilobase of transcript per million mapped reads, FPKM) for 60 carbohydrate-active enzymes and isoenzymes (divergent allelic pairs indicated GENE#_1, GENE#_2) annotated in the *F*. *cylindrus* genome sequence and proposed for a putative EPS synthesis pathway in *F. cylindrus* across six experimental phases. For gene codes, abbreviations and annotations see Table S2 and compare Fig. 6. Colour scale ranges from saturated red for highly expressed genes to saturated blue for weakly expressed genes; white indicates medium expression. A one minus Pearson's correlation distance metric was applied to cluster rows (genes) using the complete linkage method. **b** Canonical correspondence analysis triplot of the expression patterns of 60 *F. cylindrus* genes involved in a hypothetical EPS synthesis pathway (FPKM expression values, 3 groups coloured by similarity in expression pattern (see **a**), associated with temperature and salinity (centroids for phases I–VI indicated by boxed roman numerals), and diatom cell physiology and EPS production (vectors; chl *a*, chlorophyll *a* per cell; HW, CHO_HW_; HA, CHO_HA_; % of EPS in dCHO fraction; %EPS; uHB, uHA, uronic acid yield per cell in HB extraction and HA extractions; *F*_v_/*F*_m_; Glc_d_, Gal_d_, Fuc_d_, Fuc_HB_, Man_HB_ Gal_HB_, Glc_HB_, Fuc_HA_, Man_HA_, relative abundance of monosaccharides in dCHO; CHO_HB_ and CHO_HA_ fractions). Correlations between gene expression and physiological variables on CCA1 and CCA2 significant at *p* < 0.001

**Fig. 6:**
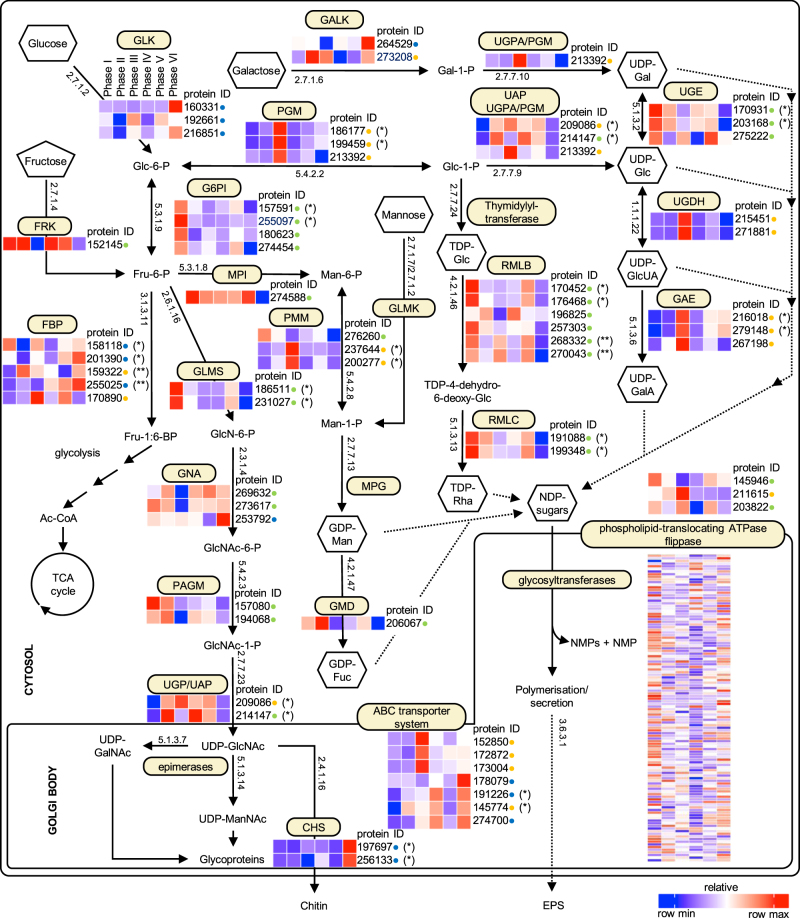
Expression of genes involved in proposed biosynthetic pathways leading to EPS production in *Fragilariopsis cylindrus* under six experimental phases. Colour scale represents absolute gene expression values (FPKM) on a relative scale per row (gene). Expression patterns for identified enzymes and isoenzymes are shown together with Joint Genome Institute (JGI) protein identifiers. Gene models belonging to divergent allelic pairs are highlighted with asterisks. To distinguish gene models associated with multiple allelic pairs encoding for a single enzyme, they are highlighted with a corresponding number of asterisks (* or **), indicating which two gene models belong to a divergent allelic pair. Green, yellow and blue dots indicate membership in one of three expression pattern clusters (see Fig. 5a). Chemical compound abbreviations: Glc-6-P glucose-6-phosphate, Glc-1-P glucose-1-phosphate, Fru-6-P fructose-6-phosphate, Fru-1:6-BP fructose-1:6-bisphosphate, UDP-Glc UDP-glucose, Gal-1-P galactose-1-phosphate, UDP-Gal UDP-galactose, Man-6-P mannose-6-phosphate, Man-1-P mannose-1-phosphate, GDP-Man GDP-mannose, GDP-Fuc GDP-fructose, TDP-Glc tyrosinediphosphate glucose, TDP-4-dehydro-6-deoxy-Glc dTDP-4-dehydro-6-deoxy-α-d-glucose, GlcN-6-P glucosamine-6-phosphate, GlcNAc-6-P *N*-acetylglucosamine-6-phosphate, GlcNAc-1-P *N*-acetylglucosamine-1-phosphate, UDP-GlcNAc UDP-*N*-acetylglucosamine, UDP-ManNAc UDP-*N*-acetylmannosamine, UDP-GalNAc UDP-*N*-acetylgalactosamine, UDP-GlcUA UDP-glucuronic acid (glucuronate), UDP-GalA UDP-galacturonicacid (galacturonate). Enzyme abbreviations: GLK glucokinase, PGM phosphoglucomutase, GALK galactokinase, UGE UDP-glucose-4-epimerase, G6PI glucose-6-phosphate isomerase, PGI phosphoglucoseisomerase, FBP fructose-bisphosphatase, PMM phosphomannomutase, MPG mannose-1-phosphateguanyltransferase, GMD GDP-mannose 4,6-dehydratase, GNA glucosamine-phosphate *N*-acetyltransferase, UDG UDP-glucose-6-dehydrogenase, GLMS glutamine-fructose-6-phosphatetransaminase, FRK fructokinase, UAP UDP-*N*-acetylglucosamine diphosphorylase, RMLB dTDP-glucose 4,6-dehydratase, PAGM phosphoacetylglucosaminemutase, RMLC dTDP-4-dehydrorhamnose 3,5-epimerase, CHS chitin synthase

Cluster analysis of expression data for 60 protein-coding genes including divergent allelic gene copies involved in pathways for the synthesis of nucleotide sugars and glycoproteins revealed three major groups (Fig. 5a). Many of the involved proteins were encoded by more than one gene, for example, the enzyme dTDP-Glc 4,6-dehydrogenase (RMLB) is encoded at four different genetic loci with two loci encoding for divergent allelic gene copies, resulting in six predicted gene models including divergent allelic gene copies (Fig. 5a, Fig. 6, Table S2). To distinguish gene models associated with multiple allelic pairs, they are highlighted with a corresponding number of asterisks, indicating which two gene models belong to a divergent allelic pair. There were significant relationships (Fig. 5b) between the absolute gene expression values, the clustering of these 60 genes including associated divergent allelic copies and the physiological responses of *F. cylindrus* across the six phases (CCA explaining 85.5% of the cumulative constrained eigenvalues, with significant correlations (*p* < 0.001) between gene expression and physiological variables). CCA axis 1 (CCA1) represented a significant gradient of increasing cell yields of CHO_HA_ uronic acids (uHA), and Fuc, Gal and Glc contents of dCHO, CHO_HB_ and CHO_HA_, and decreased EPS content of dCHO, with a positive association between CCA1 score and the period of temperature stress. CCA axis 2 (CCA2) represented a gradient of decreased photosynthetic activity (low *F*_v_/*F*_m_, high Glc content of CHO_HB_), and increased yields of CHO_HA_, uronic acids in CHO_HB_ and higher Man content in CHO_HB_ and CHO_HA_, corresponding to increases in salinity and temperature decreases to −8 °C between phases I, IV and VI (Fig. 5b). The greatest differences in physiological and gene expression response were between phase III and VI, compared to phase I, when cells had experienced longer periods of lower temperatures, agreeing with the pattern of overall gene expression (Fig. 4).

The three clusters of gene expression patterns mapped coherently to sub-elements of the reconstructed metabolic EPS pathway (Fig. 6). In phases I, II and IV, when *F. cylindrus* had the highest growth rates, the main enzymes with up-regulated expression (green dots on Fig. 6) were involved in Glc and fructose (Fru) activation pathways and conversion to the nucleotide sugars GDP-mannose (GDP-Man) and GDP-fructose (GDP-Fuc) (Fig. 5a, b, Fig. 6). Induction of RMLB and dTDP-4-dehydrorhamnose 3,5-epimerase (RMLC) in phase I and II suggests the production of TDP-Rha (Rha was found in CHO_HA_). RMLB was also induced during phases V, when increased Rha was present in CHO_HB_ and CHO_HW_. This expression cluster also included enzymes in the conversion between UDP-glucose (UDP-Glc) and UDP-galactose (UDP-Gal), and in the production pathway for amino sugars (glutamine-fructose-6-phosphatetransaminase, glucosamine-phosphate *N*-acetyl transferase (GNA), phosphoacetylglucosaminemutase) (Fig. 6).

The second expression cluster (yellow dots), including genes (phosphoglucomutase (PGM), UDP-*N*-acetylglucosamine diphosphorylase (UAP), UGPA) that catalyse the conversion of glucose-6-phosphate (Glc-6-P) to glucose-1-phosphate (Glc-1-P) potentially leading to chrysolaminarin production, and production of UDP-Glc, UDP-Gal and the uronic acid nucleotide sugars UDP-glucuronic acid (glucuronate) (UDP-GlcUA) and UDP-galacturonicacid (galacturonate) (UDP-GalA), was up-regulated during phase III (extended period at −4 °C; Fig. 6). This cluster was associated with the CCA1 gradient and with changes in cell physiology, EPS content and increased Gal content in CHO_HB_ and increased Glc content in dEPS (Fig. 5b, Fig. 3f, h). Increasing expression in uronic acid synthesis pathways, of three transmembrane phospholipid-translocating ATPases (flippases) and increased expression of ABC transporter system genes in phases III, IV and VI (Fig. 5b and 6) suggest increased production of EPS in the Golgi.

The final cluster (blue dots) contained genes highly up-regulated in phase VI, showing cells increasing their activation of intracellular Glc kinase (GLK) and passing it through fructose-6-phosphate (Fru-6-P) and fructose-1:6-bisphosphate (Fru-1:6-BP) into the tricarboxylic acid (TCA) cycle (glucose-6-phosphate isomerase (G6PI), fructose-bisphosphatase (FBP)) (Fig. 6). Increased expression of ABC transporters indicate potentially increased activity in the Golgi leading to EPS secretion. During phase VI, enzymes involved as precursors to chitin formation (GNA) were up-regulated, with chitin synthase expression markedly increased. The expression of enzymes in the main pathways for GDP-Man, GDP-Fuc, tyrosinediphosphate glucose (TDP-Glc) and TDP-Rha production were significantly reduced during phase VI.

## Discussion

EPS production by polar diatoms is a significant mechanism for survival for these important primary producers [18, 36, 69]. Data from both brines and melted ice cores (which measure different elements of the overall ice EPS pools, [16]) showed increased proportions of more chemically diverse EPS at lower temperatures. Increases in Man, Rha, Fuc, Xyl and Ara will increase the structural diversity of EPS [36, 70, 71], affording the ice crystal-influencing properties [17, 18], and formation of sticky brine channel plugs [69, 72] and protective mucilages surrounding diatom microbial cells [36, 46, 70]. Temperature and salinity is physically coupled within sea ice core profiles [73], thus as cells are incorporated within a growing ice matrix during the formation and consolidation of natural sea ice, diatoms need to adjust to decreasing temperatures and increasing salinity.

The transcriptomic and physiological results demonstrates a range of responses in the carbohydrate dynamics and EPS production of *F. cylindrus* to changing temperature and salinity, and provides a model for the production of EPS by other diatoms. Differential patterns of gene expression are part of a set of regulatory steps, including protein abundance, enzyme activation and presence of co-factors, that will change the cell metabolism and result in EPS production in diatoms [74–76]. Decreasing temperature to −4 °C had a major effect on the transcriptome of *F. cylindrus*, with increased expression of enzymes involved in RNA metabolism, translation and carbohydrate metabolism. The significant overrepresentation of metabolic processes related to RNA metabolism throughout the experimental phases II–VI agrees with previous work showing that under low temperatures, ribosomal genes and associated GO term annotations are significantly up-regulated in *F. cylindrus* to compensate for less efficient translation under low temperatures [77]. This, coupled with the strong up-regulation of an antifreeze protein (JGI protein ID 161472) under phases V and VI (Table S3), a multigene family known to be most strongly affected by lowering temperatures and increasing salinities typical for sea ice formation [78], indicates that the observed gene expression are due to decreasing temperatures and salinity.

The genome of *F. cylindrus* contains highly divergent alleles that appear adaptive to fluctuating environments [41] and divergent alleles were represented in the EPS pathway in similar proportions as they appeared in its overall genome (~25%). The ability of *F. cylindrus* to re-programme significant parts of its transcriptome, including genes involved in EPS synthesis, to acclimate to changing temperature and salinity [40], is part of a broader pattern of adaptation in this taxon to living in polar habitats.

*Fragilariopsis cylindrus* showed declines in physiological activity as temperature decreased and salinity increased. Sea ice diatoms remain physiologically active at salinities from 34 to >200, and temperatures from −1.8 °C to <−20 °C [18, 69, 79], by altering their protein expression, producing compatible solutes and antifreeze proteins [79, 80], and by the production of EPS that form barriers around cells [18, 36, 69]. Ice diatoms exhibit photo-physiological and metabolic plasticity, with a synergistic interaction between decreasing temperature and increasing salinity [42], evident from similar growth rates between phases I, II and IV, despite differences in photophysiology. The EPS and intracellular polysaccharides produced by *F. cylindrus* were similar to those of other sea ice and benthic diatom taxa [35–37, 81, 82], and to those found in the field study, with significant reprogramming evident within our reconstructed EPS production pathway, as cells responded to changes in temperature and salinity, resulting in altered EPS charactersitics.

When *F. cylindrus* was photosynthesizing and actively growing (centroids for phases I, II and IV clustered with cell growth variables in the CCA), the main genes up-regulated ('green' coded genes) were components of the pathways for Glc and Fru activation, and for conversion to Man, Fuc and Rha. Not all genes within each cluster showed identical patterns of expression (some were only highly expressed in phase I, e.g. G6PI, others more broadly, e.g. fructokinase (FRK)) as shown by the scatter in the CCA (Fig. 5b), but they all showed significant associations between gene expression, cell physiology and EPS production. Glc and Fru are products of the pentose-phosphate pathways [30], and are utilized for ATP production (glycolysis, TCA cycle), storage compounds (chrysolaminarin) or activated to make other sugars and derivatives. Strong induction of FRK and MPI in phases I, II, IV and V is indicative of activation of the Fru–Man pathway, generating mannose-6-phosphate [29, 31] which is utilised to produce the nucleotide sugars GDP-Man and GDP-Fuc (Fig. 6), and corresponds to the inclusion of Man and Fuc in EPS produced in those conditions. Increased Man and Fuc content of EPS is associated with greater surface activity and gel stiffness [71], a response to colder temperatures, altering the rheological properties of the EPS produced to provide protective cell coatings [36, 46]. We did not identify mannose-1-phosphateguanyltransferase (MPG) (that converts mannose-1-phosphate to GDP-Man), though a lack of orthologs for MPG has also been reported for the diatoms *Thalassiosira* and *Phaeodactylum* and the stramenopile macroalgae *Ectocarpus* [31]. This reaction must be catalysed by an (yet) unidentified enzyme, since Man is an important constituent of diatom EPS, particularly in lower-solubility structural EPS [35, 81]. The pathway for synthesizing UDP-*N*-acetylglucosamine, which is used for the production of glycoproteins which contribute to folding and adhesion properties in diatom EPS [33], and homologues of two cell adhesion molecules previously identified in *P. tricornutum* [33], were up-regulated in *F. cylindrus* during under temperature and salinity stress.

The conversion of Glc-6-P to Glc-1-P [29] allows for synthesis of UDP-Glc and the nucleotide sugars TDP-Glc and TDP-Rha by RMLB and RMLC. The Rha content increased in CHO_HA_ in phases I and II (and IV and V) and in ice brine EPS (Fig. 1d). We found higher proportions of Gal and Rha in CHO_HA_ in the 34 salinity treatments, with a substantial increase in Man and higher CHO_HA_ yields at higher salinity. CHO_HA_ are associated with the diatom silica frustule [82, 83], with glucuronomannans (substituted mannans with high uronic acid concentrations) the most abundant polysaccharides in diatom frustules [8, 35]. *Fragilariopsis cylindrus* and *F. curta* (also found in sea ice) have a greater Gluc-rich and Man-rich CHO_HA_ fraction compared to the ice-associated taxon *Synedropsis* [36]. These shifts reflect the metabolic flexibility of *F. cylindrus* to alter its frustule-associated polysaccharide matrix in response to the combined effects of salinity and temperature.

The most significant change in the transcriptome in phase III (−4 °C for 8 days) was up-regulation of PGM and UAP/UPGA/PGM leading to UDP-Glc, and for uronic acid precursors (UDP-GlcUa and UDP-GalA), and down-regulation of many GTs. GTs play a role in the Golgi body where the polysaccharides are constructed stepwise on the ER membranes or in the lumen of the Golgi [84], and conserved ER and Golgi body targeting motifs were identified for selected GTs in *F*. *cylindrus* including allelic variant pairs (e.g., *N*-acetylglucosaminyltransferase (JGI protein IDs 189180/142623) and chitin synthase (JGI protein IDs 197697/256133); Fig. 6). There was a decline in the %EPS in dCHO from 70% to 50% during longer exposure to −4 °C (from phase II to III, Fig. 3e) with increased production of non-EPS carbohydrates linked to physiological stress (high *σ*_PSII_ [37]). With higher salinities (typical for brine channels), cells increased the proportion of EPS in the dCHO fraction, with many of the GTs down-regulated in phase III induced in phase IV.

The third cluster of genes expressed were associated with the response of *F. cylindrus* to increased salinity, with a subset strongly expressed during phase VI. Higher salinity increases *F. cylindrus* gene expression for a range of metabolic functions, including amino-acid and carbohydrate metabolism [79], seen in phases IV, V and VI. In natural sea ice, exposure to higher salinities will only occur in parallel with declining temperatures, and our experiment was designed to simulate this simultaneous salinity and temperature stress. Previously reported pathways down-regulated in response to single salinity, or temperature modifications (e.g. energy production [79]) were not reduced in early phases, when cells up-regulated genes for RNA processing and metabolic activity to maintain growth. The diatom *Thalassiosira weissflogii* also alters its transcriptome to maintain rates of carbon metabolism and growth between salinities of 21 to 35 [85], and increased EPS production in response to salinity occurs in *Phaeodactylum tricornutum* and *Cylindrotheca closterium* [35, 86]; *F. cylindrus* [36]; and to a variable extent in *T. weissflogii* [85]. In phase IV (−4 °C, 52 salinity), *F. cylindrus* maintained its growth, with the main EPS production pathway up-regulated and various Man, Glc and uronic acid-rich EPS being produced. This ability to maintain metabolic activity under simultaneous changes in salinity and temperature is an clear adaptation by *F. cylindrus* to living in the sea ice environment [42]. RMLB and RMLC were also induced during phases IV and V, when more Rha was present in CHO_HB_ and CHO_HW_. Increases in uronic acids and Man content in EPS produced in salinity 52 will increase gel stiffness [36, 71] necessary to generate the structural mucilage barriers observed around cells in natural brine channels [17, 69].

Further low temperature stress in phases V–VI significantly reduced diatom photosynthesis and growth. Shortage of new photoassimilates would explain the increased activation of GLK, and increased expression of enzymes converting Glc through Fru-6-P and Fru-1:6-BP into the TCA cycle (G6PI, FBP) to generate ATP. Increased TCA activity is also linked to lipid production as a response to colder and more saline conditions [40]. The strong induction of almost all GTs encoded in the *F. cylindrus* genome, and several ABC transporter and flippases under the lowest temperatures and highest salinities, may reflect the need of the cells to produce protective EPS at this time. Increased expression of UDP-*N*-acetylglucosamine transferases and *α*-*N*-acetylglucosaminidases as temperatures declined and salinity increased, and high gene expression of chitin synthase (phase VI) indicate potential for chitin secretion in *F. cylindrus* EPS. Amino sugars are present in all the different EPS fractions produced by *F. cylindrus*, including in the CHO_HA_ fraction, which is closely associated with the silica frustule [36], but their functional role is unclear.

Diatoms play major roles in global primary production and biogeochemical cycles, and almost all species produce cell-associated and extracellular EPS [6, 7]. In natural sea ice, these EPS contribute to the overall carbohydrate budgets in sea ice [25]. The identification of three broad expression patterns within our reconstructed EPS production pathway, that align with physiological, biochemical and in-field measurements of EPS characteristics, provides an opportunity to investigate the environmental signals and regulators of key genes in diatom EPS production, and to elucidate the patterns of protein expression and activiation, that directly control these metabolic pathways. This raises the question whether these responses are characteristic of highly adapted polar diatoms [41] or are common features of the EPS production pathways of diatom taxa found in other environments.

## Electronic supplementary material

Supplementary Table S4

Supplementary SI detailed methods

Supplementary Figure S1

Supplementary Figure S2

Supplementary Figure S3

Supplementary Table S1

Supplementary Table S2

Supplementary Table S3
