# Supplementary material for: Identifying metabolic pathways for production of extracellular polymeric substances by the diatom Fragilariopsis cylindrus inhabiting sea ice
Source: ISME J. 2018 Jan 18;12(5):1237–51. doi: 10.1038/s41396-017-0039-z (PMC5932028; doi:10.1038/s41396-017-0039-z)
Supplement: Supplementary file 1 — Supplementary Table S4 [file 41396_2017_39_MOESM1_ESM.pdf]

Table S4. General description and mapping statistics for *Fragilariopsis cylindrus* RNA-seq data set.

**Experimental factor**	**Sample code**	**Replicate**	**Sample ID [ENA run accession]**	**Insert size**	**Total read count**	**Unique mapping (% of total)**	**Ambiguous mapping (% of total)**	**% of reads unmapped (too short)**
34 salinity, 0°C (Phase I)	Sample01_34A14C	r1	ERR2178838	191	19316820	15578518 (80.65%)	3256737 (16.86%)	2.43%
34 salinity, 0°C (Phase I)	Sample02_34B14C	r2	ERR2178839	186	18274831	14750905 (80.72%)	3063094 (16.76%)	2.45%
34 salinity, 0°C (Phase I)	Sample03_34C14C	r3	ERR2178840	191	16534534	13377420 (80.91%)	2743249 (16.59%)	2.44%
34 salinity, 2d @-4°C (Phase II)	Sample04_34A14T	r1	ERR2178841	212	18568330	14799637 (79.70%)	3261055 (17.56%)	2.68%
34 salinity, 2d @-4°C (Phase II)	Sample05_34B14T	r2	ERR2178842	202	20417032	16374211 (80.20%)	3483468 (17.06%)	2.69%
34 salinity, 2d @-4°C (Phase II)	Sample06_34C14T	r3	ERR2178843	184	19179458	15444861 (80.53%)	3279595 (17.10%)	2.33%
34 salinity, 8d @-4°C (Phase III)	Sample07_34B20T	r1	ERR2178844	200	18391657	14827506 (80.62%)	3039404 (16.53%)	2.80%
34 salinity, 8d @-4°C (Phase III)	Sample08_34C20T	r2	ERR2178845	196	20821314	16657259 (80.00%)	3504691 (16.83%)	3.12%
52 salinity, 2d @-4°C (Phase IV)	Sample09_52A14T	r1	ERR2178846	197	17584468	14155772 (80.50%)	2959097 (16.83%)	2.61%
52 salinity, 2d @-4°C (Phase IV)	Sample10_52B14T	r2	ERR2178847	190	17361202	14009084 (80.69%)	2879806 (16.59%)	2.65%
52 salinity, 2d @-4°C (Phase IV)	Sample11_52C14T	r3	ERR2178848	227	13850319	10980420 (79.28%)	2448797 (17.68%)	2.99%
52 salinity, 8d @-4°C (Phase V)	Sample12_52A20T	r1	ERR2178849	279	13074745	10308209 (78.84%)	2312330 (17.69%)	3.41%
52 salinity, 8d @-4°C (Phase V)	Sample13_52C20T	r2	ERR2178850	193	18064679	14376743 (79.58%)	3146721 (17.42%)	2.92%
52 salinity, 2d @-8°C (Phase VI)	Sample14_52B22T	r1	ERR2178851	183	18410685	14712902 (79.92%)	3170851 (17.22%)	2.79%
52 salinity, 2d @-8°C (Phase VI)	Sample15_52C22T	r2	ERR2178852	204	17092676	13632863 (79.76%)	2934582 (17.17%)	3.01%
