# Supplementary material for: Identifying metabolic pathways for production of extracellular polymeric substances by the diatom Fragilariopsis cylindrus inhabiting sea ice
Source: ISME J. 2018 Jan 18;12(5):1237–51. doi: 10.1038/s41396-017-0039-z (PMC5932028; doi:10.1038/s41396-017-0039-z)
Supplement: Supplementary file 2 — Supplementary SI detailed methods [file 41396_2017_39_MOESM2_ESM.pdf]

Supplementary Information.

Detailed description of the methods and protocols used in the field and experimental study.

Materials and Methods

Field study

EPS concentrations and composition in Antarctic Sea Ice.

Samples (sea ice brines, ice cores) were collected during two cruises to the Weddell Sea, Antarctica, in December 2004 (ISPOL) and September – October 2006 (WWOS) (Underwood *et al.* 2010; Norman *et al.* 2011), and during the Sea Ice Physics and Ecosystems Experiment (SIPEX) research expedition to Eastern Antarctica (110º – 130ºE, between September and October, 2007 (Meiners et al. 2011), thus encompassing contrasting sea ice conditions and types from winter to summer in the Weddell Sea, and from a winter to spring transition (SIPEX) (Fig. 1a).

All measurements described here were obtained from opportunistically-selected samples collected from internal sea ice brines collected with the sackhole sampling technique, and bulk sea ice from melted 10-cm-thick ice core segments (see Norman *et al.* 2011, Underwood et al. 2010 for details). The sackhole sampling technique allows internal sea ice brine to percolate into bore holes (sackholes) drilled with the stainless steel corer (Kovacs, 14 cm internal diameter) to variable depth (20 to 60 cm) below the ice surface following snow removal. Hence, data from sackhole samples refer to concentrations measured directly in sea ice brines. In the WWOS study, multiple sackhole brine samples were taken along at different locations, and subsamples taken for EPS measurements. Not all assays were conducted on each (sub)sample and some samples were lost. This is the cause for slightly different temperature values and numbers of replicates between Fig.1b and 1d. Ice cores in ISPLOL were collected using a Kovacs Mark II stainless steel ice corer (9 cm internal diameter), and a Kovacs 14 cm internal diameter corer during WWOS and SIPEX,

immediately sectioned (into 10cm sections) directly in acid-clean plastic containers, and melted (without additional liquid) in the dark at 4ºC in the onboard laboratory (Meiners *et al.,* 2011; Norman *et al.,* 2011). All samples were then filtered through pre-combusted GF/F filters (Whatman, 0.7 µm) and filters and filtrates were stored at -20°C until further analysis in the home laboratory.

Brine and ice core samples were analysed for dissolved organic carbon (DOC), total and dissolved carbohydrates and dissolved EPS (> 8 kDa molecular weight) concentrations, and EPS monosaccharide composition using methods described in Aslam et al. (2016). Data for the Weddell and East Antarctic regions were compared across four subsets based on temperature; bottom ice at the seawater interface, temperature -1.7 °C ; ice cores or brines in decreasing temperature bands of – 2 , - 4 and -8 °C (± 0.02 to 1.1 °C).

Experimental culture conditions

Axenic cultures of *Fragilariopsis cylindrus* (CCMP1102) were grown in enriched artificial seawater media (EASW) containing penicillin/streptomycin solution (Aslam *et al*., 2012) with 1.1 mmol L^{-1} nitrate concentration, at 0 °C under continuous illumination (14 µmol photons $m^{-2} s^{-1}$), and maintained in suspension by gently rotating bottles once a day. Separate stock cultures were acclimated at two salinities (34 and 52) over a three month period prior to the experiment to avoid the negative but transient impacts of acute changes in salinity on diatom photo-physiology (Krell *et al*., 2007; Aslam *et al*., 2012; Steele *et al*., 2014).

Cells (initial density of 10^5 cells ml^{-1}) were grown axenically in plastic bottles (5 L, containing 3 L of media). Treatments (a step-wise set of temperature reductions) and controls (maintained at 0 °C throughout) were established in triplicate for both salinity 34 and 52 conditions. Cultures were grown initially for 12 days at 0 °C, with a 50% volume media change at day 10 (to reduce the potential for nutrient limitation: N, P and Si concentrations in the media permit cell densities to exceed 4 x 10^6 mL^{-1} before inducing stationary phase

(Aslam *et al*., 2012), and cell densities did not exceed 1.5 x 10^6 mL^{-1} in any treatment during the experiment). The temperature reductions commenced on day 12, with designated triplicate flasks reduced to -4 °C, and these flasks, and control triplicates (maintained at the initial growing conditions of salinity 34 or 52, 0 °C), measured after 2 days (day 14, phase I, II and IV), and then after a further 6 days (day 20) at -4 °C (phases III and V), and finally (on day 22) after a further decrease to -8 °C for 2 days (phase VI). Flasks in 34 salinity were frozen solid at -8 °C (day 22) and so samples were not taken, and the 54 salinity flasks froze *after* 2 days at -8 °C, terminating the temperature reduction sequence. All samples were taken before decreasing the temperature on the corresponding days. Subsamples (180 to 230 ml) from each replicate flask on d 0, 10, 14, 20, 22, for measurements of cell density (haemocytometer), cell photophysiology, carbohydrate content, and biochemical composition (see below). The same triplicate flasks under the temperature-reduction treatment were repeat-sampled on the later days (phase II to III; phases IV, V and VI). Intrinsic growth rate (μ d^{-1}) was calculated from the logistic growth equation. Chlorophyll a concentration was determined at each time point (29 mL subsamples filtered through precombusted GF/F filters, filters macerated in 4 ml of 100% methanol saturated with anhydrous $MgCO_3$, with a for 24 h extraction at 4 °C (Aslam *et al*., 2012).

Cell photophysiology, carbohydrate and EPS production and composition

Cell PSII photochemistry was determined using a Satlantic FIRe fluorometer (Satlantic Inc. Halifax, Canada). Samples (3mL) were dark adapted for 30 min at 0 °C and measurements made immediately (at room temperature), measuring a four step transient determining single turnover (ST) excitation from a 100 μs pulse, ST relaxation over 500 ms, multiple turnover (MT) excitation from a 600 ms pulse, and MT relaxation over 1 s. Twenty sequential measurements were averaged to increase the signal to noise ratio (Suggett *et al*., 2008). Using FIREPRO software (Satlantic Inc.), the maximum PSII photochemical

efficiency (Fv/Fm) and functional absorbance cross-section of photosystem II (σ_{PSII}, nm^2 $RCII^{-1}$) were calculated (Aslam *et al*. 2012).

Carbohydrate fraction sampling and extraction.

Diatom EPS were fractionated into: (a) dissolved carbohydrate (dCHO) fractions containing both polymeric (dissolved EPS, dEPS) and non-polymeric (lower molecular weight) carbohydrates secreted by cells into the culture media and (b) particulate carbohydrates, using established sequential extractions (Aslam *et al*., 2012). Subsamples of culture (150 to 200 ml) were centrifuged at 3500*g* for 15 min. The supernatant was used for determination of dissolved carbohydrates (dCHO) and dissolved EPS. Supernatant amples were dialysed overnight (at 20 °C) through 8 kDa dialysis tubing against ultra pure water (18.2 MΩ cm, MilliQ) and freeze-dried. Freeze-dried samples were re-dissolved in ultra-pure water and divided into 3 aliquots of 1 to 4 mL each. One aliquot was used to determine carbohydrate concentration and monosaccharide content (described later); 2 aliquots were used to isolate dEPS with different solubility, by precipitation with 30% and 70% ethanol, termed $dEPS_{complex}$ and dEPS respectively (Underwood *et al*., 2013).

Pellets containing cells and associated particulate carbohydrates were sequentially extracted (Aslam *et al*., 2012). A hot water-extracted carbohydrate (CHO_{HW}) fraction (mainly intracellular storage polysaccharides) was obtained by macerating pellets in 0.5 M NaCl (salinity 30), incubation at 100 °C for 1 h followed by centrifugation (3500 *g*, 15 min). A hot bicarbonate-extracted (CHO_{HB}) fraction (solubilisng gelatinous and water-insoluble EPS) was obtained by incubating the remaining cell pellet with 0.5M $NaHCO_3$ at 100 °C for 1 h followed by centrifugation (3500 *g*, 15 min). Finally a hot alkali extraction (CHO_{HA}) with 1 M NaOH and 0.2 M $NaBH_4$ at 100 °C for 1 h followed by centrifugation (3500 *g*, 15 min) liberated EPS associated with the silica frustules. An aliquot (1 mL) of each extracted fraction (CHO_{HW}, CHO_{HB}, CHO_{HA}) was used to determine carbohydrate concentration

(phenol sulphuric acid assay). The remaining CHO_{HW}, CHO_{HB} and CHO_{HA} extracts were dialysed overnight (as above), then freeze-dried for measurement of uronic acids and determination of monosaccharide composition (HW, HB and HA). Carbohydrate concentrations in each fraction were determined using a modified phenol sulphuric acid assay (Aslam *et al*., 2012) with glucose as a standard. Uronic acids were determined by standard carbazole assay (Aslam *et al*., 2012) with glucuronic acid as standard. Neutral monosaccharide composition was determined by gas chromatography linked with mass spectroscopy (GC-MS) (Underwood *et al*., 2010; Aslam *et al*., 2012). Polysaccharides and standards were hydrolyzed, saponified, and reduced to the corresponding alditols. Alditols were acetylated, and monosaccharide separation was carried out using a RT-2330 column. Inositol was used as the internal standard (Underwood *et al*., 2010; Aslam *et al*., 2012).

RNA extraction, RNA-seq library preparation and sequencing.

Subsamples (200-250 ml) of culture were filtered onto Isopore Polycarbonate filters (1.2 µm, 47 mm, Millipore) using a vacuum filtration system. Filters were wrapped in aluminium foil, immediately frozen in liquid nitrogen and stored at -80 °C. Total RNA was extracted using guanididium thiocyanate-phenol-chloroform (TRI Reagent, Sigma-Aldrich, St. Louis, MO, USA) extraction (Chomczynski and Sacchi, 2006), followed by DNase I (Quiagen, Hilden, Germany) treatment (1 h at 37 °C) and purification using RNeasy MinElute Cleanup Kits (Quiagen, Hilden, Germany) according to the manufacturer's instructions. Purity of RNA was checked using a NanoDrop (Thermo Fisher Scientific, Waltham, MA, USA). RNA integrity was confirmed using denaturing formaldehyde RNA gels in MOPS buffer containing 1.2% agarose and 2% formaldehyde.

Library preparation and RNA sequencing was performed at the Earlham Institute (formerly The Genome Analysis Centre TGAC, Norwich, UK). After initial RNA quality checks using Qubit fluorometric assays (ThermoFisher Scientific, Waltham, MA, USA) and

an Agilent Bioanalyzer equipped with a Nano kit (Agilent, Santa Clara, CA, USA), libraries were constructed according to the Illumina TruSeq RNA protocol 15026495 Rev. B (Illumina, San Diego, CA, USA) using a Sciclone automated liquid handling workstation (Perkin Elmer, Waltham, MA, USA). 1µg of total RNA was purified for polyA-containing mRNA using oligo-dT attached beads and fragmented. RNA fragments primed with random hexamers were reverse transcribed into first strand cDNA, followed by second strand synthesis to generate double stranded cDNA and cDNA end repair. The 3' ends of cDNAs were adenylated and multiple indexing adapters containing complementary 'T' overhangs were ligated to the fragments. The ligated products were size selected using Beckman Coulter XP beads (Beckman Coulter, Brea, CA, USA) to remove free adapters and adapters ligated to one another. Samples were enriched for fragments with adapters ligated on both cDNA ends using PCR with primers annealing to the ends of adapters. The insert size of ~200bp of the sample libraries was verified using a PerkinElmer GX equipped with a high sensitivity DNA chip (PerkinElmer), and concentrations were determined using a high sensitivity Qubit fluorometric assay and qPCR. The indexed cDNA libraries were normalized to 10 nM using Elution Buffer (Quiagen, Hilden, Germany) and pooled in equal volumes. Prior to hybridisation to the flow cell, cDNA library pools were spiked with PhiX control v3 (Illumina). The flow cell was clustered using the TruSeq Paired End Cluster Generation Kit v3 and sequencing was performed on a HiSeq 2000 (Illumina). Each library pool was run in a single lane for 50 cycles of each paired end read. Sequencing reads in bcl format were de-multiplexed based on the 6 bp Illumina index using CASSAVA, allowing for a one base-pair mismatch per library, and converted to FASTQ format using bcl2fastq.

Read mapping and transcript quantification.

Preparation of 50 bp paired end libraries and RNA sequencing with a HiSeq2000 instrument (Illumina, San Diego, CA, USA) was performed at the Earlham Institute

(Norwich, UK). After initial RNA quality checks, multiplexed cDNA libraries were constructed, with each library pool run in a single lane. Sequencing reads were de-multiplexed using CASAVA (Illumina, San Diego, CA, USA), allowing for a one base-pair mismatch per library. Sequencing data was cleaned using Trim Galore! v0.4.4 (Krueger, 2017) with FastQC v0.11.5 (Andrews, 2017) and Cutadapt v1.14 (Martin, 2011). Results were summarized in a single report using MultiQC v1.2 (Ewels *et al.* 2016). The RNA-seq aligner STAR v2.5.3a (Dobin et al. 2013) was used to align reads to the *F. cylindrus* genome assembly v1.0 (Fracy1_assembly_scaffolds.fasta.gz; http://genome.jgi-psf.org/Fracy1/) allowing for a maximum of two mismatches (`--outFilterMismatchNmax 2`) to ensure stringent alignment of reads to divergent alleles and allele-specific RNA-seq analysis (Mock *et al.*, 2017). The programme featureCounts (Liao *et al.* 2014) implemented in the Bioconductor R subread package was used to count reads.

Differential expression and Gene Ontology enrichment analysis

Differential gene expression analysis was performed using edgeR (Robinson *et al.*, 2010). Weakly expressed genes were filtered from the data set and analysis was performed on transcriptionally active regions. A gene model was considered transcriptionally active if its sum of counts (row) that mapped to the model in one or more libraries (columns) was greater than 0. The default trimmed mean of M values (TMM) normalisation method (Robinson *et al.* 2010) was used to calculate the effective library size. After estimating genewise (tagwise) dispersions and fitting negative binomial models, respective edgeR features were used to perform a multidimensional scaling (MDS) analysis of the top set of 5000 genes with highest biological variation (top=5000 option) and statistical testing for differentially expressed genes was performed using the generalized linear models (glm) feature. To detect differentially expressed genes, pair-wise multiple comparisons were performed between experimental treatments using the glm likelihood ratio test (McCarthy *et al.*, 2012) and p-values were

corrected for multiple testing (Benjamini and Hochberg, 1995).

A functional gene ontology (GO) analysis was performed on differentially expressed genes ($p < 0.05$) using goseq (Young *et al*., 2010), and enriched GO terms were identified for either up- or down-regulated transcripts as established from pair-wise comparisons using edgeR. To perform the analysis and account for a gene lengths selection bias, the GO term annotations associated with each gene and gene lengths were extracted from the *F. cylindrus* filtered models 1 annotation file (available at http://genome.jgi.doe.gov/Fracy1/Fracy1.download.ftp.html) using customised Perl scripts. Analysis was performed according to the goseq method, estimating a probability weighing function for the data and using the implemented Wallenius non-central hyper-geometric distribution (Wallenius, 1963). Enriched GO terms with $p < 0.05$ were selected. Results were summarized by removing redundant GO terms and visualised in semantic similarity-based scatterplots using REVIGO (http://revigo.irb.hr/ (Supek *et al*., 2011).

Identification and hierarchical clustering analysis of carbohydrate-related proteins

Fragilariopsis cylindrus genes (Fracy1_GeneModels_FilteredModels2_aa.fasta.gz) encoding for carbohydrate-active enzymes were identified based on homology with biochemically characterized proteins from the Carbohydrate-Active enZYmes (CAZy) Database (www.cazy.org, Lombard *et al*., 2014). After normalisation of read counts to mean fragments per kilobase of transcript per million mapped reads (FPKM) expression values using the rpkm function of edgeR, a hierarchical clustering analysis of was performed for 194 carbohydrate-active enzyme identified. A one minus Pearson correlation distance metric and the average linking method was applied to cluster genes (rows) using R software (RDevelopment Core Team, 2015), and results visualised using the Heatmap function of the Bioconductor ComplexHeatmap package (Gu *et al*., 2016).

Reconstruction of a hypothetical EPS pathway map

A draft reconstruction of carbohydrate metabolic pathways leading to EPS production was generated based on the annotated set of carbohydrate-active enzymes and manual curation of metabolic genes from the most recent annotation of the *F. cylindrus* genome (available at http://genome.jgi.doe.gov/Fracy1/Fracy1.home.html) using Gene Ontology (GO), KEGG pathways and clusters of eukaryotic orthologous groups of proteins (KOG (Tatusov *et al*., 2003) information. Additionally, using canonical polysaccharide biosynthesis pathways (Fabris *et al*., 2012; Michal, 2014) and bibliographic resources phylogenetically close organisms (Kroth *et al*., 2008; Michel *et al*., 2010; Donot *et al*., 2012; Fabris *et al*., 2012; Willis *et al*., 2014), metabolic genes were collected using BLAST (Altschul *et al*., 1997) searches, and targeted searches for E.C. numbers (Nomenclature Committee, 1992) and keywords. All candidate carbohydrate metabolic genes were collected in a spreadsheet that was used for manual curation and refinement. For the reconstruction of the pathway map, we analyzed all collected metabolic genes for the presence of signal peptides using SignalP (Nielsen 2017), selecting only proteins that are predicted to be cytosolic and lack any conserved plastid (ASAF) or mitochondrial targeting sequences. The EPS pathway was assembled using a general protocol for genome-scale metabolic reconstructions (Thiele and Palsson, 2010) starting from the canonical polysaccharide pathways and metabolic reactions catalysed by identified gene products were connected based on E.C. numbers as informed by KEGG and BRENDA biochemical reaction databases and mapped to the experimental gene expression data during sea ice formation.

Statistical analysis

Statistical analyses were conducted using SPSS® 18.0 and Minitab v.13.3 (Minitab Inc). Significant differences were determined using t-test and analysis of variance (ANOVA, with Tukey post hoc tests). Normality of samples was checked, but the assumption of normality could not be rejected in any case. All statistically significant differences quoted are

at $p \leq 0.05$ (two-tailed). Monosaccharide compositional data for carbohydrate fractions were analysed using ANOSIM and SIMPER (Primer v.6, Plymouth, U.K.). Canonical correspondence analysis (CCA) was used to extract the major significant relationships present between the physiological, biochemical and transcriptome datasets using the data values for each treatment (using MVSP v3.1, Kolvec Ltd, N. Wales, U.K.).

Data Availability

RNA-seq data are available in the ArrayExpress database www.ebi.ac.uk/arrayexpress) under accession number E-MTAB-5153.
