# Supplementary material for: Identifying metabolic pathways for production of extracellular polymeric substances by the diatom Fragilariopsis cylindrus inhabiting sea ice
Source: ISME J. 2018 Jan 18;12(5):1237–51. doi: 10.1038/s41396-017-0039-z (PMC5932028; doi:10.1038/s41396-017-0039-z)
Supplement: Supplementary file 3 — Supplementary Figure S1 [file 41396_2017_39_MOESM3_ESM.pdf]

Figure S1 a

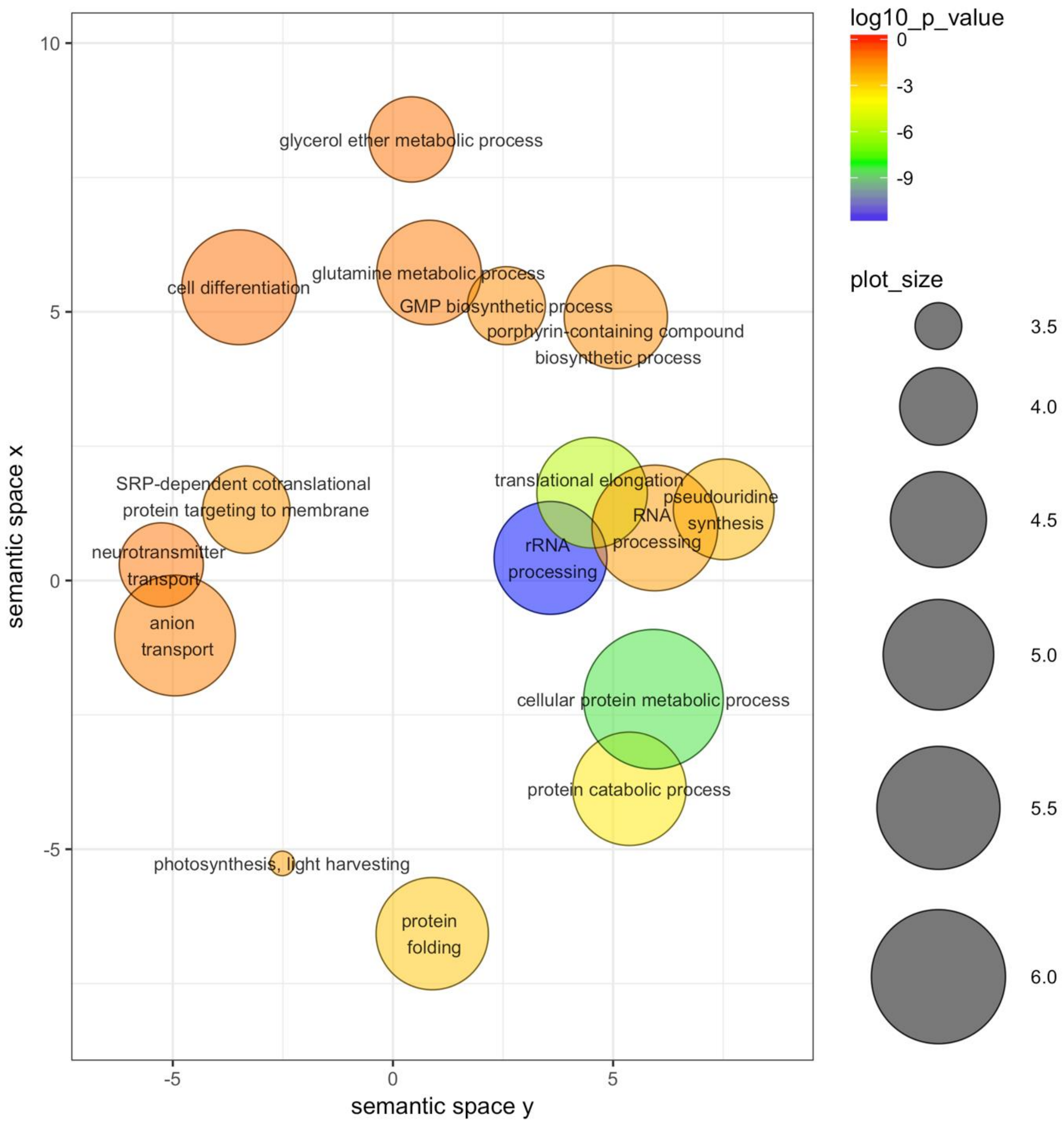

log10_p_value
0
-3
-6
-9
plot_size
3.5
4.0
4.5
5.0
5.5
6.0
glycerol ether metabolic process
cell differentiation
glutamine metabolic process
GMP biosynthetic process
porphyrin-containing compound biosynthetic process
SRP-dependent cotranslational protein targeting to membrane
neurotransmitter transport
anion transport
translational elongation
pseudouridine synthesis
RNA processing
rRNA processing
cellular protein metabolic process
protein catabolic process
photosynthesis, light harvesting
protein folding
semantic space x
semantic space y
10
5
0
-5

Figure S1 b

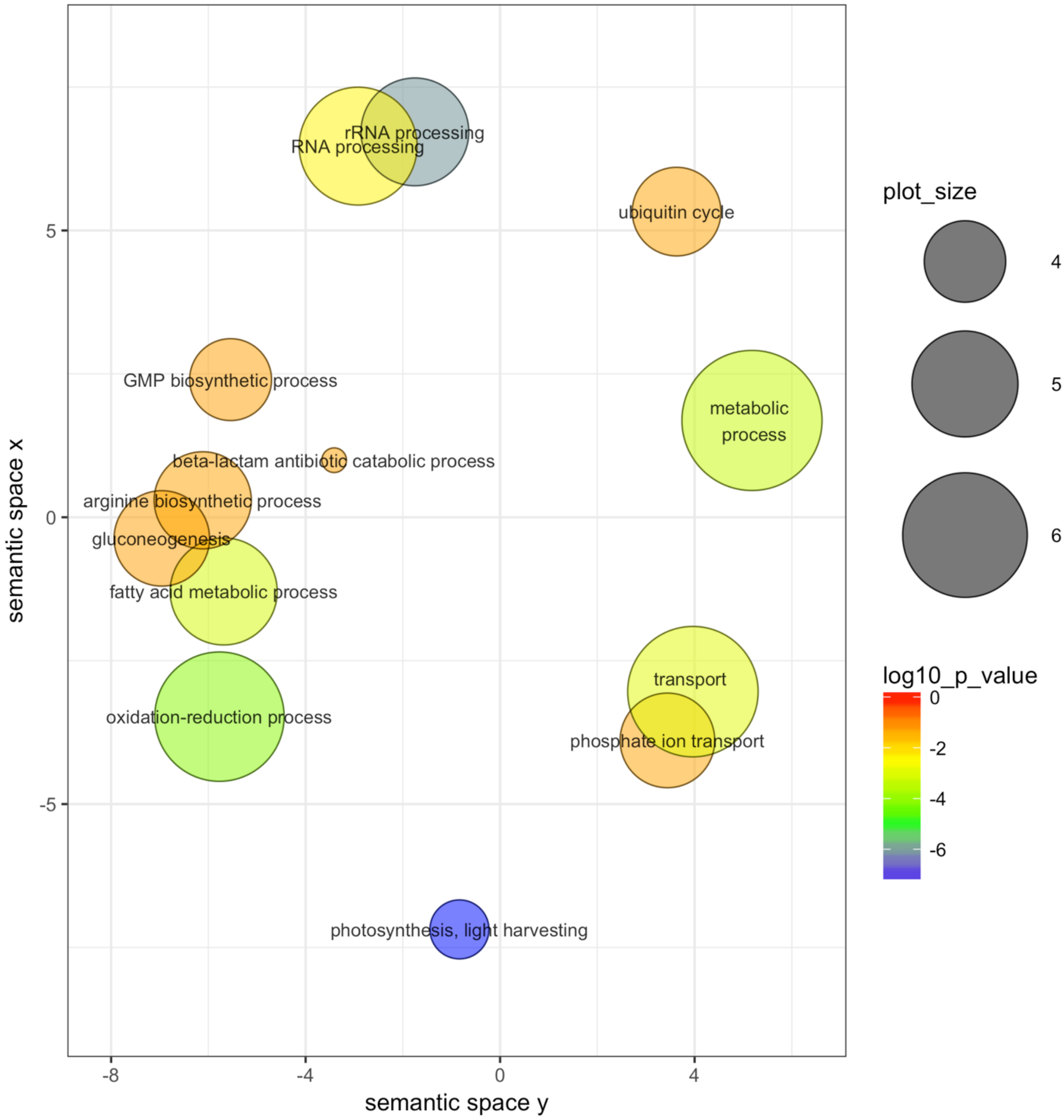
rRNA processing
RNA processing
ubiquitin cycle
GMP biosynthetic process
metabolic process
beta-lactam antibiotic catabolic process
arginine biosynthetic process
gluconeogenesis
fatty acid metabolic process
transport
oxidation-reduction process
phosphate ion transport
photosynthesis, light harvesting
semantic space x
semantic space y
5
0
-5
-8
-4
0
4
plot_size
4
5
6
log10_p_value
0
-2
-4
-6

Figure S1 c

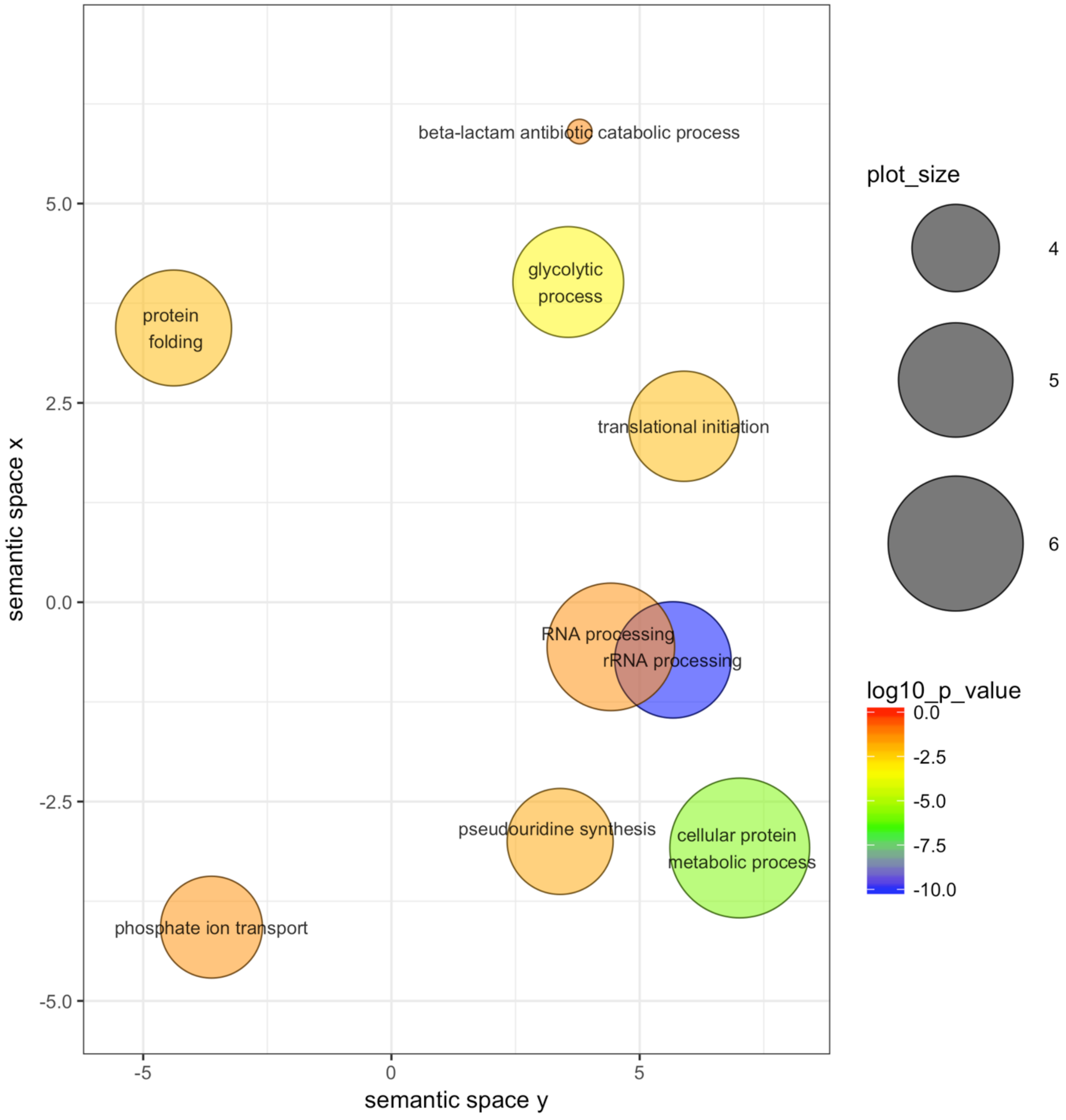
beta-lactam antibiotic catabolic process
glycolytic process
protein folding
translational initiation
RNA processing
rRNA processing
pseudouridine synthesis
cellular protein metabolic process
phosphate ion transport
5.0
2.5
0.0
-2.5
-5.0
semantic space x
-5
0
5
semantic space y
plot_size
4
5
6
log10_p_value
0.0
-2.5
-5.0
-7.5
-10.0

Figure S1 d

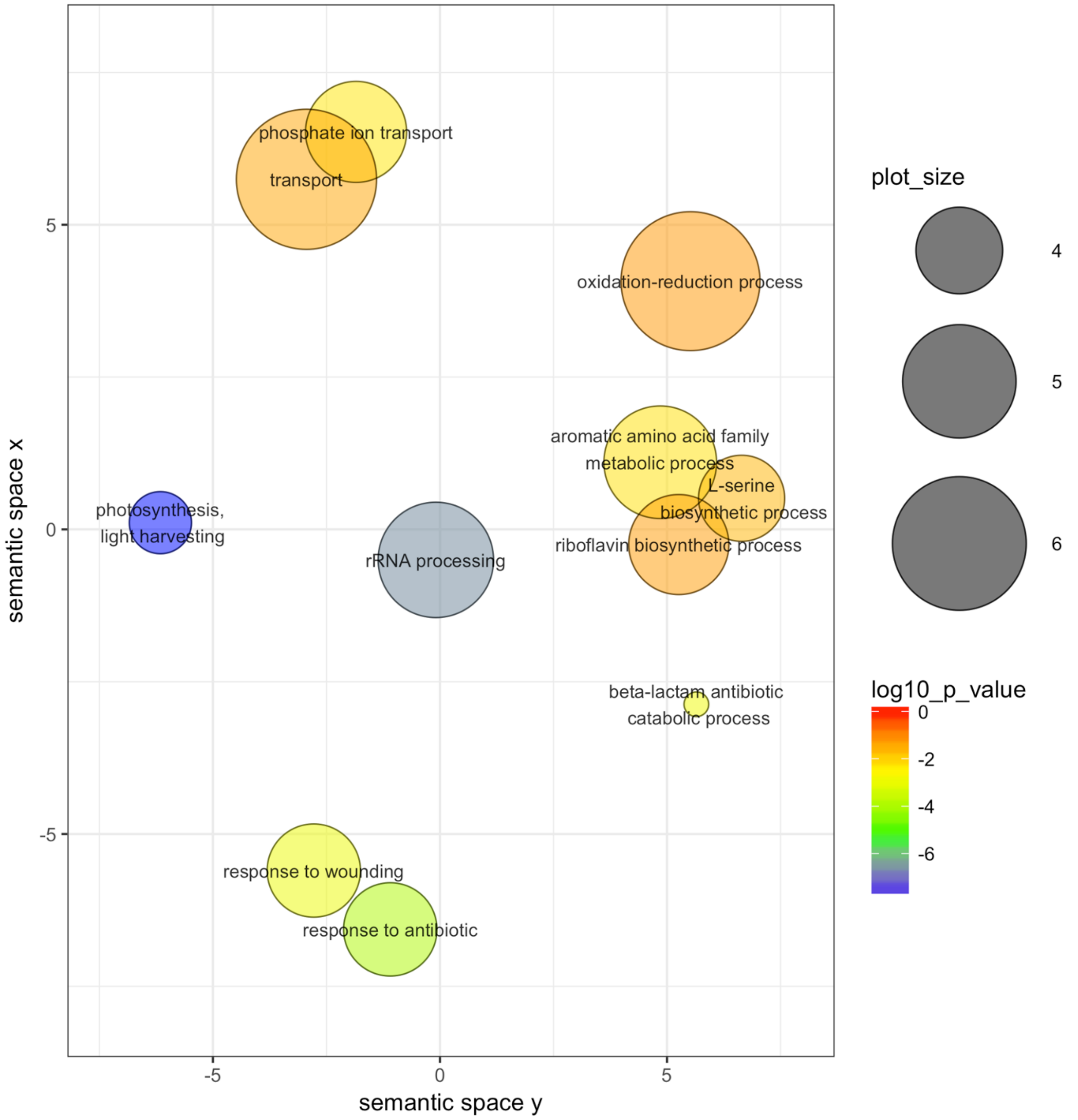

phosphate ion transport
transport
oxidation-reduction process
aromatic amino acid family
metabolic process
L-serine
biosynthetic process
riboflavin biosynthetic process
photosynthesis,
light harvesting
rRNA processing
beta-lactam antibiotic
catabolic process
response to wounding
response to antibiotic
semantic space x
semantic space y
5
0
-5
plot_size
4
5
6
log10_p_value
0
-2
-4
-6

Figure S1 e

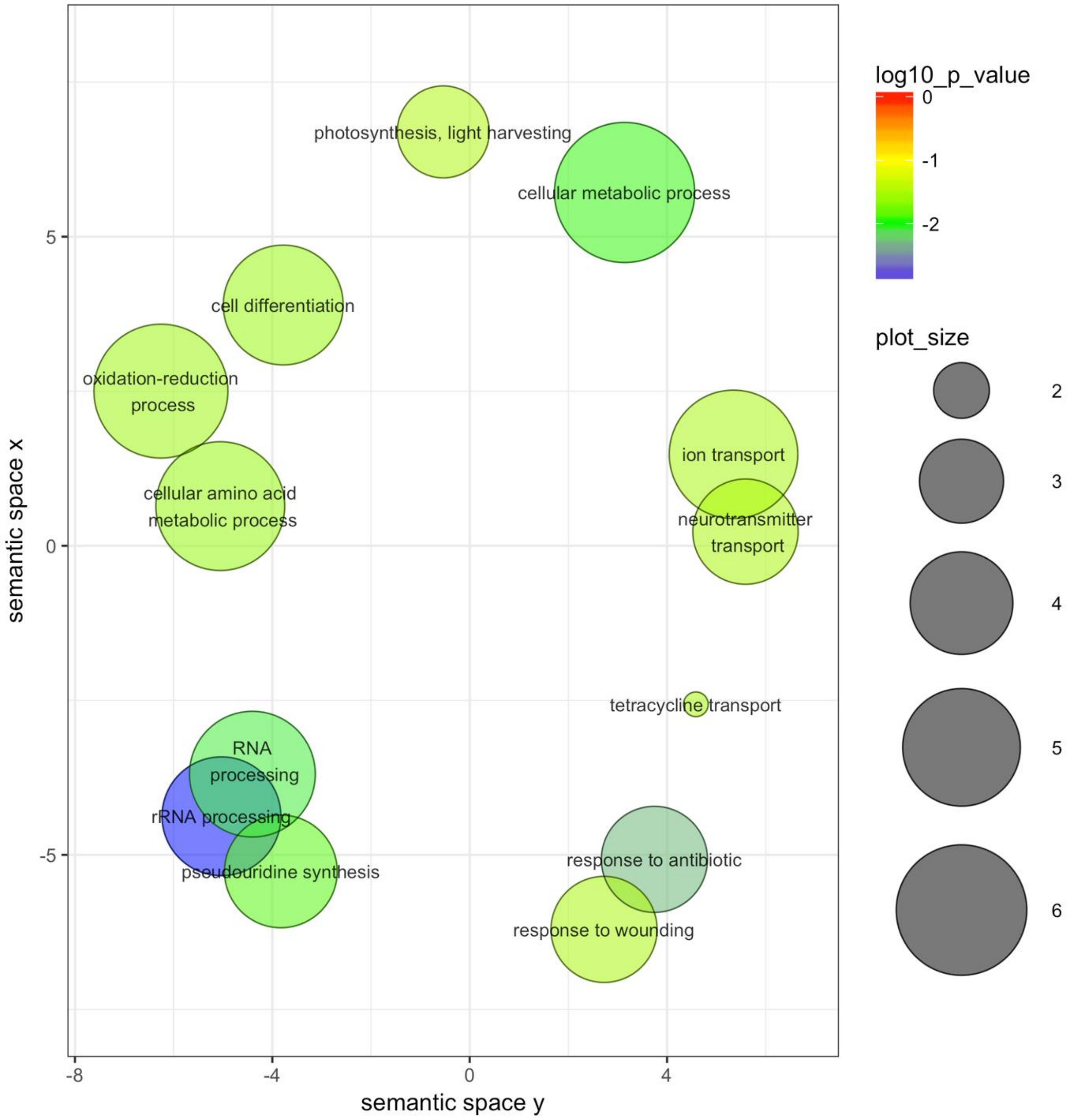

Figure S1 a – e: ReViGO scatterplots showing enriched Biological Process GO terms of all differentially expressed genes in *Fragilariopsis cylindrus* significantly up-regulated ($p < 0.05$) during phase II (**a**), III (**b**), IV (**c**), V (**d**) and VI (**e**) relative to open seawater phase I. Overrepresented GO terms (Wallenius approximation, Benjamini-Hochberg adjusted $p < 0.05$) among significantly up-regulated genes (GLM likelihood ratio test, $p < 0.05$) were determined using the goseq Bioconductor R package (Young et al., 2010). A non-redundant GO term set was plotted in a two-dimensional space by applying a multidimensional scaling procedure so that more semantically similar GO terms are closer in the plot using the ReViGO Web server (http://revigo.irb.hr/). Allowed similarity was set to 0.5 (small) with all other parameters set to default. The bubble color indicates significance levels and size indicates the frequency of the GO term in the underlying Gene Ontology Annotation (UniProt-GOA) Database.
