# Supplementary material for: Identifying metabolic pathways for production of extracellular polymeric substances by the diatom Fragilariopsis cylindrus inhabiting sea ice
Source: ISME J. 2018 Jan 18;12(5):1237–51. doi: 10.1038/s41396-017-0039-z (PMC5932028; doi:10.1038/s41396-017-0039-z)
Supplement: Supplementary file 4 — Supplementary Figure S2 [file 41396_2017_39_MOESM4_ESM.pdf]

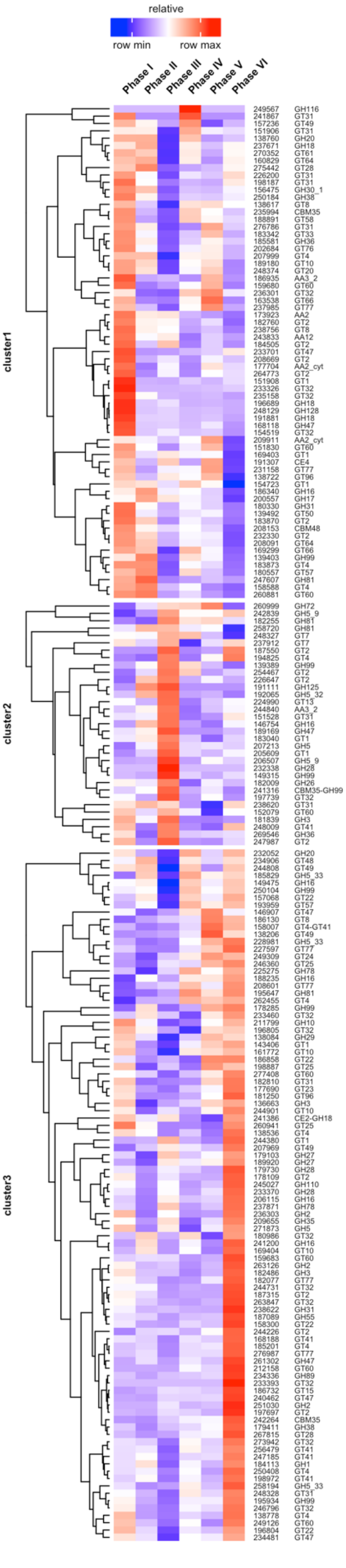
relative
row min
row max
Phase I
Phase II
Phase III
Phase IV
Phase V
Phase VI
cluster1
cluster2
cluster3
249567 GH116
241867 GT31
157236 GT49
151906 GT31
138760 GH20
237671 GH18
270352 GT61
160829 GT64
275442 GT28
226200 GT31
198187 GT31
156475 GH30_1
250184 GH38
138617 GT8
235994 CBM35
188891 GT58
276786 GT31
183342 GT33
185581 GH36
202684 GT76
207999 GT4
189180 GT10
248374 GT20
186935 AA3_2
159680 GT60
236301 GT32
163538 GT66
237985 GT77
173923 AA2
182760 GT2
238756 GT8
243833 AA12
184505 GT2
233701 GT47
208669 GT2
177704 AA2_cyt
264773 GT2
151908 GT1
233326 GT32
235158 GT32
196689 GH18
248129 GH128
191881 GH18
168118 GH47
154519 GT32
209911 AA2_cyt
151830 GT60
169403 GT1
191307 CE4
231158 GT77
138722 GT96
154723 GT1
186340 GH16
200557 GH17
180330 GH31
139492 GT50
183870 GT2
208153 CBM48
232330 GT2
208091 GT64
169299 GT66
139403 GH99
183873 GT4
180557 GT57
247607 GH81
158588 GT4
260881 GT60
260999 GH72
242839 GH5_9
182255 GH81
258720 GH81
248327 GT7
237912 GT7
187550 GT2
194825 GT4
139389 GH99
254467 GT2
226647 GT2
191111 GH125
192065 GH5_32
224990 GT13
244840 AA3_2
151528 GT31
146754 GH16
189169 GH47
183040 GT1
207213 GH5
205609 GT1
206507 GH5_9
232338 GH28
149315 GH99
182009 GH26
241316 CBM35-GH99
197739 GT32
238620 GT31
152079 GT60
181839 GH3
248009 GT41
269546 GH36
247987 GT2
232052 GH20
234906 GT48
244808 GT49
185829 GH5_33
149475 GH16
250104 GH99
157068 GT22
193959 GT57
146907 GT47
186130 GT8
158007 GT4-GT41
138206 GT49
228981 GH5_33
227597 GT77
249309 GT24
246360 GT25
225275 GH78
188235 GH16
208601 GT77
195647 GH81
262455 GT4
178285 GH99
233460 GT32
211799 GH10
196805 GT32
138084 GH29
143406 GT1
161772 GT10
186858 GT22
198887 GT25
277408 GT60
182810 GT31
177690 GT23
181250 GT96
136663 GH3
244901 GT10
241386 CE2-GH18
260941 GT25
138536 GT4
244380 GT1
207969 GT49
179103 GH27
189920 GH27
179730 GH28
178109 GT2
245027 GH110
233370 GH28
206115 GH16
237871 GH78
236303 GH2
209655 GH35
271873 GH5
180986 GT32
241200 GH16
169404 GT10
159683 GT60
263126 GH2
182486 GH3
182077 GT77
244731 GT32
187315 GT2
263847 GT32
238622 GH31
187089 GH55
158300 GT22
244226 GT2
168188 GT41
185201 GT4
276987 GT77
261302 GH47
212158 GT60
234336 GH89
233393 GT32
186732 GT15
240462 GT47
251030 GH2
197697 GT2
242264 CBM35
179411 GH38
267815 GT28
273942 GT32
256479 GT41
247185 GT41
184113 GH1
250408 GT4
198972 GT41
258194 GH5_33
248328 GT31
195934 GH99
246796 GT32
138778 GT4
249126 GT60
196804 GT22
234481 GT47

Figure S2: Hierarchical clustering analysis of expression values (mean fragments per kilobase of transcript per million mapped reads, FPKM) for 194 carbohydrate-active enzymes encoding genes annotated in the *Fragilariopsis cylindrus* genome sequence (http://genome.jgi.doe.gov/Fracy1/Fracy1.home.html) using the CArbohydrate-Active enZYmes (CAZy) database across 6 experimental phases with stepwise decreasing temperatures and increasing salinity. Color scale ranges from saturated red for highly expressed genes to saturated blue for weakly expressed genes; white indicates medium expression. The heatmap was generated using ComplexHeatmap (Gu *et al.*, 2016) using k-means clustering with a one minus Pearson correlation distance metric and average linkage method to cluster rows (genes).
