# Supplementary material for: Identifying metabolic pathways for production of extracellular polymeric substances by the diatom Fragilariopsis cylindrus inhabiting sea ice
Source: ISME J. 2018 Jan 18;12(5):1237–51. doi: 10.1038/s41396-017-0039-z (PMC5932028; doi:10.1038/s41396-017-0039-z)
Supplement: Supplementary file 5 — Supplementary Figure S3 [file 41396_2017_39_MOESM5_ESM.pdf]

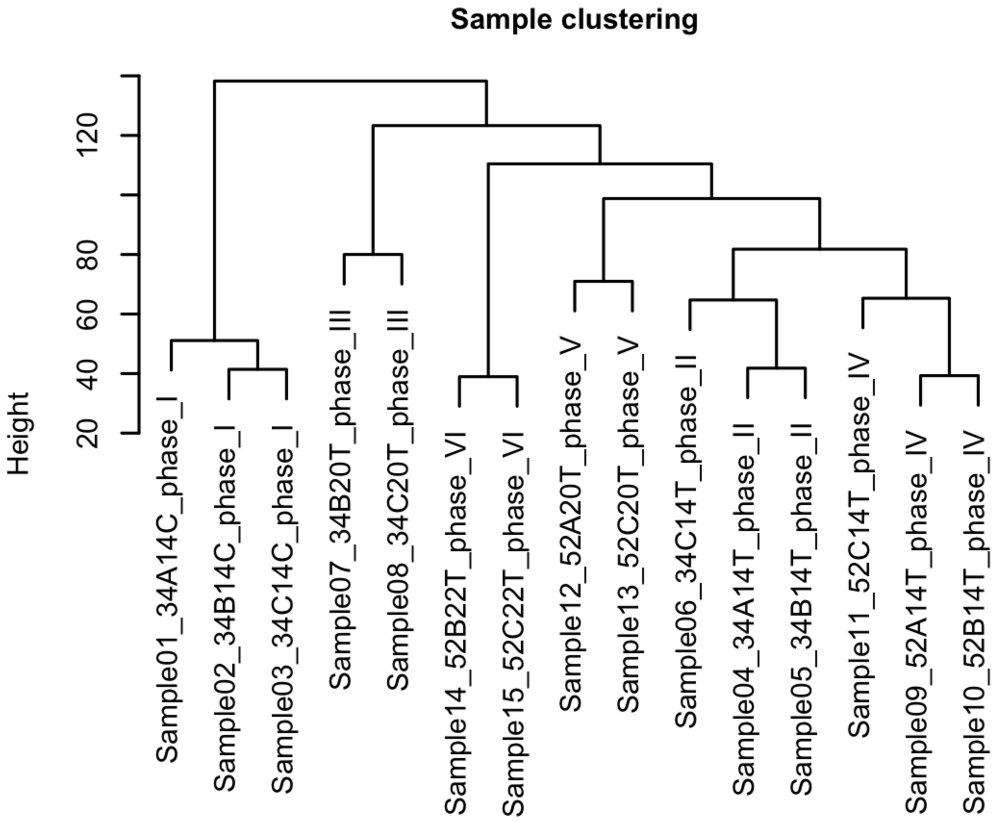

Figure S3. Tree showing sample clustering of all analyzed samples. Hierarchical clustering analysis was performed on log2 transformed expression values (mean fragments per kilobase of transcript per million mapped reads, FPKM) using the hclust function in R applying the average linkage method
