# Supplementary material for: Identifying metabolic pathways for production of extracellular polymeric substances by the diatom Fragilariopsis cylindrus inhabiting sea ice
Source: ISME J. 2018 Jan 18;12(5):1237–51. doi: 10.1038/s41396-017-0039-z (PMC5932028; doi:10.1038/s41396-017-0039-z)
Supplement: Supplementary file 6 — Supplementary Table S1 [file 41396_2017_39_MOESM6_ESM.pdf]

Table S1. Protein identifiers (ID) and annotations for 195 carbohydrate-active enzymes identified in the genome of the sea ice diatom *Fragilariopsis cylindrus* based on homology with biochemically characterized proteins from the Carbohydrate-Active enZYmes (CAZy) Database (www.cazy.org) and their average gene expression (FPKM) values under six experimental phases of temperature and salinity.

**proteinId**	**description**	**modelnotes**	**defline**	**phase_I**	**phase_II**	**phase_III**	**phase_IV**	**phase_V**	**phase_VI**
243833	AA12		Auxilliary Activities Family 12 protein	43.57	23.78	27.13	7.77	18.54	12.62
241386	CE2-GH18		Carbohydrate Esterase Family 2 / Glycoside Hydrolase Family 18 protein	24.46	20.52	22.97	16.62	13.63	26.41
191307	CE4		Carbohydrate Esterase Family 4 protein	53.95	38.74	52.80	36.91	57.68	26.70
241316	CBM35-GH99		Carbohydrate-Binding Module Family 35 / Glycoside Hydrolase Family 99 protein	9.11	12.23	23.56	7.07	10.75	4.36
235994	CBM35		Carbohydrate-Binding Module Family 35 protein	23.17	15.01	11.16	20.32	17.98	15.62
242264	CBM35		Carbohydrate-Binding Module Family 35 protein	0.29	0.25	0.48	0.62	0.64	1.35
208153	CBM48		Carbohydrate-Binding Module Family 48 protein	42.19	33.89	30.34	32.97	30.16	21.12
173923	AA2		Class II peroxidase	184.55	134.74	137.36	97.58	86.84	71.40
177704	AA2_cyt		Cytochrome c peroxidase	28.61	9.02	17.24	9.83	20.17	15.37
209911	AA2_cyt		Cytochrome c peroxidase	81.29	75.19	68.99	77.16	93.25	51.37
244840	AA3_2	splicing fragment;	GMC oxidoreductase	0.78	1.04	1.57	0.46	0.77	0.96
186935	AA3_2		GMC oxidoreductase	90.62	60.01	61.84	57.10	79.39	57.46
184113	GH1		Glycoside Hydrolase Family 1 protein	32.41	36.28	25.19	33.71	30.10	41.95
211799	GH10	splicing fragment;	Glycoside Hydrolase Family 10 protein	9.23	8.11	6.75	7.08	8.22	8.93
245027	GH110		Glycoside Hydrolase Family 110 protein	2.99	1.73	3.03	1.91	2.39	5.10
249567	GH116		Glycoside Hydrolase Family 116 protein	0.00	0.00	0.00	0.01	0.00	0.00
191111	GH125	fragment N-term;	Glycoside Hydrolase Family 125 protein	0.00	0.04	0.05	0.00	0.00	0.00
248129	GH128	splicing fragment;	Glycoside Hydrolase Family 128 protein	7.56	1.57	1.84	1.31	1.42	1.01
186340	GH16		Glycoside Hydrolase Family 16 protein	19.36	23.94	17.38	18.14	15.81	9.32
188235	GH16	fragment N-term;	Glycoside Hydrolase Family 16 protein	2.69	5.23	3.90	5.51	5.58	6.71
146754	GH16		Glycoside Hydrolase Family 16 protein	10.05	12.66	13.89	10.08	9.41	11.35
241200	GH16		Glycoside Hydrolase Family 16 protein	16.20	19.37	16.07	18.14	15.74	22.49
149475	GH16	fragment N-term;	Glycoside Hydrolase Family 16 protein	44.46	45.77	32.19	47.95	43.61	48.35
206115	GH16	fragment N-term;	Glycoside Hydrolase Family 16 protein	28.06	23.58	31.99	27.51	28.46	40.24
200557	GH17	splicing fragment;	Glycoside Hydrolase Family 17 protein	58.55	81.90	64.14	54.30	45.98	11.11
196689	GH18		Glycoside Hydrolase Family 18 protein	26.16	2.87	4.93	0.91	1.26	0.53
237671	GH18	splicing fragment;	Glycoside Hydrolase Family 18 protein	7.80	8.65	4.67	7.62	6.52	7.48
191881	GH18	fragment N-term / C-term;	Glycoside Hydrolase Family 18 protein	15.62	3.16	3.28	2.10	2.60	0.98

proteinId	description	modelnotes	defline	phase_I	phase_II	phase_III	phase_IV	phase_V	phase_VI
236303	GH2		Glycoside Hydrolase Family 2 protein	4.87	2.76	4.09	3.06	3.16	5.61
263126	GH2	splicing fragment;	Glycoside Hydrolase Family 2 protein	3.57	2.73	2.87	2.32	2.74	5.24
251030	GH2		Glycoside Hydrolase Family 2 protein	2.44	2.44	2.69	2.47	2.52	3.80
138760	GH20		Glycoside Hydrolase Family 20 protein	12.23	12.24	8.17	11.96	9.57	10.19
232052	GH20		Glycoside Hydrolase Family 20 protein	4.99	4.10	3.24	5.48	4.39	5.38
182009	GH26		Glycoside Hydrolase Family 26 protein	6.45	5.89	8.47	3.86	6.02	5.16
179103	GH27		Glycoside Hydrolase Family 27 protein	2.16	1.35	2.85	2.10	2.41	3.16
189920	GH27	fragment C-term;	Glycoside Hydrolase Family 27 protein	6.70	4.37	10.15	8.77	9.77	13.16
232338	GH28	fragment N-term;	Glycoside Hydrolase Family 28 protein	0.00	0.00	0.06	0.00	0.00	0.00
233370	GH28		Glycoside Hydrolase Family 28 protein	10.51	7.44	11.23	8.48	10.39	16.56
179730	GH28	splicing fragment;	Glycoside Hydrolase Family 28 protein	2.70	2.17	2.78	2.56	2.29	4.15
138084	GH29		Glycoside Hydrolase Family 29 protein	1.35	1.21	0.95	1.18	1.37	1.32
182486	GH3		Glycoside Hydrolase Family 3 protein	6.84	5.88	5.86	5.28	5.75	8.21
181839	GH3	splicing fragment;	Glycoside Hydrolase Family 3 protein	5.93	4.71	6.73	3.36	3.31	3.65
136663	GH3	fragment N-term;	Glycoside Hydrolase Family 3 protein	2.50	1.22	1.72	1.96	2.42	2.66
156475	GH30_1		Glycoside Hydrolase Family 30 protein	34.63	31.66	20.16	25.26	24.85	27.70
180330	GH31		Glycoside Hydrolase Family 31 protein	63.43	53.34	47.69	52.53	49.98	44.16
238622	GH31		Glycoside Hydrolase Family 31 protein	0.86	0.72	0.74	0.70	0.69	1.40
209655	GH35		Glycoside Hydrolase Family 35 protein	25.44	15.94	28.75	11.57	22.07	38.03
269546	GH36	fragment N-term;	Glycoside Hydrolase Family 36 protein	30.92	15.86	39.41	23.64	24.43	25.34
185581	GH36	fragment N-term;	Glycoside Hydrolase Family 36 protein	5.55	4.85	3.65	4.41	4.81	4.39
250184	GH38	splicing fragment;	Glycoside Hydrolase Family 38 protein	4.29	3.57	2.69	3.24	3.20	3.39
179411	GH38		Glycoside Hydrolase Family 38 protein	3.60	3.32	3.56	3.66	3.80	4.54
261302	GH47		Glycoside Hydrolase Family 47 protein	15.07	16.76	14.77	17.01	15.85	23.97
168118	GH47	fragment N-term / C-term;	Glycoside Hydrolase Family 47 protein	79.63	50.06	50.61	48.53	53.66	43.62
189169	GH47		Glycoside Hydrolase Family 47 protein	4.28	4.78	6.68	3.44	3.42	3.90
192065	GH5_32		Glycoside Hydrolase Family 5 protein	9.72	13.72	15.77	9.00	9.37	7.96
258194	GH5_33	fragment C-term;	Glycoside Hydrolase Family 5 protein	1.08	0.64	0.73	1.35	0.91	2.21
185829	GH5_33	fragment N-term;	Glycoside Hydrolase Family 5 protein	15.12	21.43	13.34	22.69	19.92	21.07
228981	GH5_33	fragment N-term;	Glycoside Hydrolase Family 5 protein	3.68	2.78	3.03	4.79	6.10	6.96
206507	GH5_9	splicing fragment;	Glycoside Hydrolase Family 5 protein	19.07	20.32	64.54	18.47	33.71	25.61
242839	GH5_9	fragment C-term;	Glycoside Hydrolase Family 5 protein	0.00	0.04	0.14	0.09	0.09	0.12
271873	GH5	splicing fragment;	Glycoside Hydrolase Family 5 protein	6.22	4.21	5.72	3.68	5.27	6.72

proteinId	description	modelnotes	defline	phase_I	phase_II	phase_III	phase_IV	phase_V	phase_VI
207213	GH5		Glycoside Hydrolase Family 5 protein	5.95	12.47	21.55	11.18	11.76	10.64
187089	GH55		Glycoside Hydrolase Family 55 protein	8.15	7.90	6.93	7.59	7.69	12.10
260999	GH72		Glycoside Hydrolase Family 72 protein	37.64	49.98	57.78	58.70	66.84	37.62
237871	GH78		Glycoside Hydrolase Family 78 protein	5.24	3.03	7.96	3.86	4.90	10.43
225275	GH78		Glycoside Hydrolase Family 78 protein	5.44	3.91	5.67	7.26	6.17	7.25
182255	GH81	fragment N-term;	Glycoside Hydrolase Family 81 protein	54.14	41.12	148.24	111.57	117.20	87.02
258720	GH81	splicing fragment;	Glycoside Hydrolase Family 81 protein	1.07	1.39	1.38	1.09	1.14	0.52
247607	GH81	splicing fragment;	Glycoside Hydrolase Family 81 protein	5.60	6.05	4.28	4.45	4.65	4.42
195647	GH81	fragment N-term;	Glycoside Hydrolase Family 81 protein	7.53	7.87	8.61	11.90	10.56	12.00
234336	GH89		Glycoside Hydrolase Family 89 protein	13.38	11.47	13.20	13.61	14.39	27.81
195934	GH99	splicing fragment;	Glycoside Hydrolase Family 99 protein	3.24	1.96	1.12	2.85	2.83	5.01
139403	GH99		Glycoside Hydrolase Family 99 protein	7.59	8.72	5.70	7.65	7.16	5.75
149315	GH99	fragment N-term;	Glycoside Hydrolase Family 99 protein	16.43	14.64	46.85	19.04	14.84	20.07
178285	GH99		Glycoside Hydrolase Family 99 protein	32.06	35.45	32.11	33.26	36.49	37.35
139389	GH99	fragment C-term;	Glycoside Hydrolase Family 99 protein	8.10	10.93	15.24	12.07	8.75	6.28
250104	GH99	splicing fragment;	Glycoside Hydrolase Family 99 protein	3.30	3.45	1.80	3.96	3.40	3.90
143406	GT1	fragment N-term;	Glycosyltransferase Family 1 protein	30.17	20.51	16.12	26.21	30.66	33.34
151908	GT1	fragment N-term;	Glycosyltransferase Family 1 protein	22.78	1.58	1.26	3.61	1.94	4.73
154723	GT1	fragment C-term;	Glycosyltransferase Family 1 protein	9.10	8.86	7.72	9.42	8.39	4.41
247210	GT1	fragment C-term;	Glycosyltransferase Family 1 protein	0.00	0.00	0.00	0.00	0.00	0.00
183040	GT1	fragment C-term;	Glycosyltransferase Family 1 protein	3.88	4.19	4.99	3.68	3.00	3.74
244380	GT1		Glycosyltransferase Family 1 protein	11.06	11.38	18.01	11.18	9.45	21.26
205609	GT1	fragment N-term;	Glycosyltransferase Family 1 protein	4.08	6.68	15.25	7.09	5.89	8.10
169403	GT10		Glycosyltransferase Family 10 protein	21.36	15.61	18.13	16.63	16.80	9.06
169404	GT10	fragment N-term;	Glycosyltransferase Family 10 protein	8.08	9.46	7.76	9.03	7.44	11.14
244901	GT10	splicing fragment;	Glycosyltransferase Family 10 protein	1.06	0.47	0.88	0.67	1.02	1.44
161772	GT10	fragment N-term;	Glycosyltransferase Family 10 protein	47.83	39.78	34.37	45.76	46.46	48.20
189180	GT13		Glycosyltransferase Family 13 protein	7.34	6.66	4.12	6.17	6.56	4.81
224990	GT15		Glycosyltransferase Family 15 protein	2.85	2.46	3.74	2.16	2.56	2.83
186732	GT2	splicing fragment;	Glycosyltransferase Family 2 protein	20.79	22.02	25.53	25.07	23.96	42.31
187550	GT2	splicing fragment;	Glycosyltransferase Family 2 protein	16.79	16.76	26.59	16.22	17.32	25.36
182760	GT2		Glycosyltransferase Family 2 protein	5.14	3.98	3.92	3.06	3.55	2.49
197697	GT2		Glycosyltransferase Family 2 protein	7.44	7.15	8.57	8.23	7.32	15.47

proteinId	description	modelnotes	defline	phase_I	phase_II	phase_III	phase_IV	phase_V	phase_VI
247987	GT2	splicing fragment;	Glycosyltransferase Family 2 protein	1.11	0.29	1.50	0.35	0.31	0.60
232330	GT2		Glycosyltransferase Family 2 protein	62.78	53.98	39.51	47.42	46.30	27.76
254467	GT2		Glycosyltransferase Family 2 protein	31.21	40.01	56.78	48.42	34.96	38.98
208669	GT2		Glycosyltransferase Family 2 protein	23.27	17.93	19.01	17.28	19.27	18.32
226647	GT2	splicing fragment;	Glycosyltransferase Family 2 protein	5.99	8.36	9.24	6.73	7.49	6.72
264773	GT2		Glycosyltransferase Family 2 protein	11.33	6.03	7.91	6.00	8.14	7.53
244226	GT2		Glycosyltransferase Family 2 protein	0.01	0.16	0.17	0.09	0.25	0.58
187315	GT2		Glycosyltransferase Family 2 protein	48.74	46.17	42.90	43.59	43.62	57.65
183870	GT2		Glycosyltransferase Family 2 protein	25.33	21.48	14.81	18.72	17.00	13.70
178109	GT2	splicing fragment;	Glycosyltransferase Family 2 protein	18.52	15.31	17.35	16.17	16.07	21.92
184505	GT20		Glycosyltransferase Family 20 protein	48.53	33.75	42.20	29.15	34.37	30.01
248374	GT22	splicing fragment;	Glycosyltransferase Family 22 protein	29.33	26.53	18.43	24.92	27.33	20.81
157068	GT22	splicing fragment;	Glycosyltransferase Family 22 protein	10.95	13.01	7.78	13.62	11.90	14.60
158300	GT22	splicing fragment;	Glycosyltransferase Family 22 protein	6.08	5.00	4.92	5.82	5.47	9.73
196804	GT22	splicing fragment;	Glycosyltransferase Family 22 protein	9.35	7.89	6.16	8.07	7.59	9.77
186858	GT23	splicing fragment;	Glycosyltransferase Family 23 protein	5.85	4.97	5.39	4.53	7.57	7.61
177690	GT24	fragment N-term;	Glycosyltransferase Family 24 protein	119.24	77.96	65.02	91.03	104.08	152.48
249309	GT25		Glycosyltransferase Family 25 protein	12.50	8.57	14.50	18.34	23.20	21.47
246360	GT25	splicing fragment;	Glycosyltransferase Family 25 protein	2.22	1.25	3.52	3.29	5.44	6.32
260941	GT25	fragment N-term;	Glycosyltransferase Family 25 protein	7.20	5.66	4.24	4.29	3.98	7.01
198887	GT28		Glycosyltransferase Family 28 protein	49.14	15.01	31.53	19.74	56.55	49.29
275442	GT28		Glycosyltransferase Family 28 protein	15.25	16.52	11.34	12.58	12.98	14.32
267815	GT31	fragment N-term;	Glycosyltransferase Family 31 protein	15.36	12.31	13.50	19.36	19.90	35.89
226200	GT31	splicing fragment;	Glycosyltransferase Family 31 protein	13.05	10.57	6.97	10.12	9.52	11.56
241867	GT31	splicing fragment;	Glycosyltransferase Family 31 protein	0.06	0.00	0.00	0.07	0.00	0.00
182810	GT31	fragment N-term / C-term;	Glycosyltransferase Family 31 protein	88.40	67.08	68.91	70.32	86.99	97.41
151528	GT31	splicing fragment;	Glycosyltransferase Family 31 protein	13.80	14.40	22.63	8.67	10.16	16.49
248328	GT31	fragment C-term;	Glycosyltransferase Family 31 protein	43.84	38.08	31.67	46.12	40.79	55.67
151906	GT31	fragment N-term / C-term;	Glycosyltransferase Family 31 protein	5.40	5.38	3.58	6.04	5.08	4.93
276786	GT31	fragment N-term / C-term;	Glycosyltransferase Family 31 protein	13.75	12.51	9.91	10.71	13.58	11.60
238620	GT31		Glycosyltransferase Family 31 protein	10.80	11.44	10.97	9.04	7.54	11.00
198187	GT32	splicing fragment;	Glycosyltransferase Family 32 protein	3.38	2.65	2.03	2.31	2.26	2.76

proteinId	description	modelnotes	defline	phase_I	phase_II	phase_III	phase_IV	phase_V	phase_VI
180986	GT32		Glycosyltransferase Family 32 protein	36.94	39.37	36.63	39.12	34.58	40.55
244731	GT32	splicing fragment;	Glycosyltransferase Family 32 protein	8.20	8.20	6.57	6.97	6.84	11.53
236301	GT32	splicing fragment;	Glycosyltransferase Family 32 protein	1.07	0.69	0.67	1.14	1.29	0.96
235158	GT32		Glycosyltransferase Family 32 protein	3.91	2.05	1.34	1.28	1.35	1.10
233460	GT32		Glycosyltransferase Family 32 protein	10.21	12.65	10.19	6.92	12.47	15.05
233393	GT32		Glycosyltransferase Family 32 protein	0.00	0.00	0.00	0.00	0.00	0.03
263847	GT32	splicing fragment;	Glycosyltransferase Family 32 protein	7.39	6.38	5.71	5.53	5.93	10.77
154519	GT32	fragment N-term / C-term;	Glycosyltransferase Family 32 protein	46.01	18.49	22.51	18.07	19.02	12.20
233326	GT32	splicing fragment;	Glycosyltransferase Family 32 protein	35.66	4.99	4.97	5.42	6.06	7.36
197739	GT32	fragment C-term;	Glycosyltransferase Family 32 protein	2.16	1.89	2.81	1.84	2.18	1.80
246796	GT32		Glycosyltransferase Family 32 protein	11.00	7.90	6.84	10.24	9.49	14.61
196805	GT32		Glycosyltransferase Family 32 protein	1.44	1.23	0.90	0.55	1.31	1.37
273942	GT33		Glycosyltransferase Family 33 protein	7.67	7.40	5.87	8.29	7.41	10.70
183342	GT4-GT41	fragment N-term;	Glycosyltransferase Family 4 / Glycosyltransferase Family 41 protein	24.77	18.94	12.65	17.13	22.54	15.98
158007	GT4	splicing fragment;	Glycosyltransferase Family 4 protein	0.46	0.23	0.32	0.59	1.38	1.09
138536	GT4	fragment C-term;	Glycosyltransferase Family 4 protein	27.88	26.69	23.40	25.26	23.05	30.83
207999	GT4		Glycosyltransferase Family 4 protein	72.06	71.89	40.32	65.23	66.04	55.48
262455	GT4		Glycosyltransferase Family 4 protein	0.30	0.58	0.59	0.99	0.92	1.00
185201	GT4		Glycosyltransferase Family 4 protein	19.86	21.71	13.95	22.62	23.84	45.34
158588	GT4		Glycosyltransferase Family 4 protein	15.10	15.60	12.77	13.62	12.07	11.53
138778	GT4	fragment N-term / C-term;	Glycosyltransferase Family 4 protein	7.92	6.59	6.31	7.03	6.80	8.52
183873	GT4		Glycosyltransferase Family 4 protein	13.06	13.07	7.92	10.78	10.22	8.36
194825	GT4	fragment C-term;	Glycosyltransferase Family 4 protein	37.06	35.94	49.57	36.51	43.19	49.75
250408	GT41	fragment N-term;	Glycosyltransferase Family 41 protein	13.51	14.09	10.79	14.46	11.65	19.57
247185	GT41	splicing fragment;	Glycosyltransferase Family 41 protein	10.20	10.84	8.21	10.09	10.20	13.42
248009	GT41	splicing fragment;	Glycosyltransferase Family 41 protein	12.47	9.31	13.45	9.59	8.97	12.45
256479	GT41		Glycosyltransferase Family 41 protein	6.62	6.82	4.40	7.46	6.48	9.66
198972	GT41		Glycosyltransferase Family 41 protein	4.30	5.24	2.71	4.96	3.82	7.97
168188	GT47	splicing fragment;	Glycosyltransferase Family 47 protein	11.88	11.09	7.97	11.95	11.00	20.31
233701	GT47	splicing fragment;	Glycosyltransferase Family 47 protein	11.75	1.76	2.01	2.97	3.67	6.19
234481	GT47		Glycosyltransferase Family 47 protein	12.62	11.25	7.71	11.88	11.47	14.10
240462	GT47	splicing fragment;	Glycosyltransferase Family 47 protein	0.53	0.50	0.78	0.77	0.79	2.10

proteinId	description	modelnotes	defline	phase_I	phase_II	phase_III	phase_IV	phase_V	phase_VI
146907	GT48	fragment N-term / C-term;	Glycosyltransferase Family 48 protein	48.57	41.45	44.30	50.73	55.83	48.35
234906	GT49		Glycosyltransferase Family 49 protein	15.27	17.00	11.90	15.97	14.77	16.19
157236	GT49	splicing fragment;	Glycosyltransferase Family 49 protein	4.24	3.76	3.11	4.49	2.61	3.23
207969	GT49		Glycosyltransferase Family 49 protein	13.35	15.81	18.05	16.74	15.59	21.65
244808	GT49	splicing fragment;	Glycosyltransferase Family 49 protein	8.00	8.40	4.48	8.37	6.06	8.40
138206	GT50	splicing fragment;	Glycosyltransferase Family 50 protein	0.14	0.16	0.19	0.30	0.67	0.42
139492	GT57	fragment C-term;	Glycosyltransferase Family 57 protein	38.89	28.44	25.45	27.94	28.97	22.08
193959	GT57		Glycosyltransferase Family 57 protein	15.41	16.95	12.51	18.97	17.53	18.80
180557	GT58		Glycosyltransferase Family 58 protein	30.61	33.12	18.14	28.78	23.53	19.51
188891	GT60		Glycosyltransferase Family 60 protein	59.78	42.43	36.22	54.56	50.45	46.58
277408	GT60	splicing fragment;	Glycosyltransferase Family 60 protein	11.09	10.32	8.54	10.07	11.49	13.40
159680	GT60		Glycosyltransferase Family 60 protein	57.14	39.17	41.28	46.87	52.64	39.34
212158	GT60	fragment C-term;	Glycosyltransferase Family 60 protein	42.04	42.15	41.55	52.54	47.74	80.56
260881	GT60		Glycosyltransferase Family 60 protein	6.22	6.35	4.95	5.29	4.93	4.66
159683	GT60	fragment C-term;	Glycosyltransferase Family 60 protein	58.09	60.70	60.10	48.71	52.65	100.72
152079	GT60	fragment C-term;	Glycosyltransferase Family 60 protein	1.29	1.07	1.34	1.12	0.72	1.15
151830	GT60	fragment C-term;	Glycosyltransferase Family 60 protein	34.67	28.41	22.49	27.39	32.72	19.28
249126	GT61		Glycosyltransferase Family 61 protein	1.87	1.39	1.09	1.62	1.19	2.15
270352	GT64		Glycosyltransferase Family 64 protein	22.30	21.62	15.78	20.83	18.43	20.45
160829	GT64	fragment N-term / C-term;	Glycosyltransferase Family 64 protein	15.23	15.83	7.23	13.67	9.93	13.53
208091	GT66		Glycosyltransferase Family 66 protein	136.68	125.89	99.83	111.78	109.63	82.91
169299	GT66	fragment N-term;	Glycosyltransferase Family 66 protein	56.73	54.88	40.89	55.52	50.70	42.73
163538	GT7	fragment N-term / C-term;	Glycosyltransferase Family 7 protein	42.41	27.15	26.52	36.71	47.54	27.41
248327	GT7	fragment C-term;	Glycosyltransferase Family 7 protein	16.71	16.51	18.64	18.29	15.26	12.23
237912	GT76		Glycosyltransferase Family 76 protein	3.93	3.70	4.11	3.22	3.74	3.98
202684	GT77		Glycosyltransferase Family 77 protein	37.80	32.33	26.51	32.07	33.42	29.71
208601	GT77		Glycosyltransferase Family 77 protein	2.24	2.75	2.90	3.53	3.02	3.81
227597	GT77	fragment N-term;	Glycosyltransferase Family 77 protein	10.49	7.48	7.39	11.25	14.70	18.82
182077	GT77	splicing fragment;	Glycosyltransferase Family 77 protein	8.16	7.18	6.19	6.39	7.69	10.96
231158	GT77		Glycosyltransferase Family 77 protein	32.75	23.85	27.90	28.28	34.14	19.53
276987	GT77		Glycosyltransferase Family 77 protein	5.27	5.60	4.51	5.58	5.57	8.35
237985	GT8		Glycosyltransferase Family 8 protein	19.48	15.31	12.30	17.26	21.09	14.61

proteinId	description	modelnotes	defline	phase_I	phase_II	phase_III	phase_IV	phase_V	phase_VI
186130	GT8		Glycosyltransferase Family 8 protein	1.34	1.86	2.22	2.38	3.94	3.41
238756	GT8	splicing fragment;	Glycosyltransferase Family 8 protein	4.48	3.57	3.46	2.88	3.09	2.66
138617	GT96	fragment N-term;	Glycosyltransferase Family 96 protein	8.73	6.59	4.07	7.96	8.09	7.41
138722	GT96		Glycosyltransferase Family 96 protein	7.39	5.30	6.73	6.28	7.63	3.07
181250	AA1	fragment N-term;	Multicopper oxidase	24.11	14.16	14.36	16.46	21.84	33.31
