# Supplementary material for: Identifying metabolic pathways for production of extracellular polymeric substances by the diatom Fragilariopsis cylindrus inhabiting sea ice
Source: ISME J. 2018 Jan 18;12(5):1237–51. doi: 10.1038/s41396-017-0039-z (PMC5932028; doi:10.1038/s41396-017-0039-z)
Supplement: Supplementary file 7 — Supplementary Table S2 [file 41396_2017_39_MOESM7_ESM.pdf]

Table S2. Protein identifiers (ID), enzyme annotations, abbreviation codes and identification numbers (coloured by gene expression cluster, see Fig. 5) for 60 protein-coding genes and divergent alleles involved in proposed EPS production pathways (Fig. 6). The list is sorted according to identification numbers given in Fig. 5. Divergent allelic pairs identified in the *Fragilariopsis cylindrus* genome are indicated with abbreviation codes in parentheses, according to the naming scheme GENE#_1 and GENE#_2 for allele 1 and 2, respectively.

**Protein ID**	**Enzyme**	**abbrev. code**	**Number in Fig. 5**
203822	phospholipid-translocating P-type ATPase, flippase	FLP	1
275222	UDP-glucose 4-epimerase	UGE	2
274588	mannose-6-phosphate isomerase	MPI	3
270043	bifunctional dTDP-glucose 4,6-dehydratase/UDP-glucuronate decarboxylase	RMLB (RMLB2_2)	4
268332	bifunctional dTDP-glucose 4,6-dehydratase/UDP-glucuronate decarboxylase	RMLB (RMLB2_1)	5
274454	glucose-6-phosphate isomerase	G6PI	6
170452	dTDP-glucose 4,6-dehydratase	RMLB (RMLB3_1)	7
199348	bifunctional dTDP-4-dehydrorhamnose 3,5-epimerase/dTDP-4-dehydrorhamnose reductase	RMLC (RMLC1_2)	8
157591	glucose-6-phosphate isomerase	G6PI (G6PI1_1)	9
186511	glutamine-fructose-6-phosphate transaminase (isomerizing)	GLMS (GLMS1_1)	10
231027	glutamine-fructose-6-phosphate transaminase (isomerizing)	GLMS (GLMS1_2)	11
176468	dTDP-glucose 4,6-dehydratase	RMLB (RMLB3_2)	12
180623	glucose-6-phosphate isomerase	G6PI	13
255097	glucose-6-phosphate isomerase	G6PI (G6PI1_2)	14
214147	UDP-N-acetylglucosamine diphosphorylase/ UTP--glucose OR galactose-1-phosphate uridylyltransferase	UAP/UGPA/PGM (UAP1_2)	15
269632	glucosamine-phosphate N-acetyltransferase	GNA	16
196825	dTDP-glucose 4,6-dehydratase	RMLB	17
152145	fructokinase	FRK	18
194068	phosphoacetylglucosamine mutase	PAGM	19
145946	phospholipid-translocating P-type ATPase, flippase	FLP	20
273617	glucosamine-phosphate N-acetyltransferase	GNA	21
206067	GDP-mannose 4,6-dehydratase	GMD	22
157080	phosphoacetylglucosamine mutase	PAGM	23
191088	bifunctional dTDP-4-dehydrorhamnose 3,5-epimerase/dTDP-4-dehydrorhamnose reductase	RMLC (RMLC1_1)	24
203168	UDP-glucose 4-epimerase	UGE (UGE1_2)	25
257303	bifunctional dTDP-glucose 4,6-dehydratase/UDP-	RMLB	26

	glucuronate decarboxylase		
170931	UDP-glucose 4-epimerase	UGE (UGE1_1)	27
267260	phosphomannomutase	PMM	28
273208	galactokinase	GALK	29
145774	ABC transporter	ABC (ABC_2)	30
159322	fructose-1,6-bisphosphatase	FBP (FBP2_1)	31
172872	ABC transporter	ABC	32
216018	UDP-glucuronate 4-epimerase	GAE (GAE3_1)	33
279148	UDP-glucuronate 4-epimerase	GAE (GAE3_2)	34
152850	chaperone ABC transporter	ABC	35
173004	ABC transporter	ABC	36
186177	phosphoglucomutase	PGM (PGM1_1)	37
199459	phosphoglucomutase	PGM (PGM1_2)	38
211615	phospholipid-translocating P-type ATPase, flippase	FLP	39
200277	phosphomannomutase	PMM (PMM2_2)	40
267198	UDP-glucuronate 4-epimerase	GAE	41
237644	phosphomannomutase	PMM (PMM2_1)	42
213392	UTP-glucose-1-phosphate uridylyltransferase/phosphoglucomutase fusion protein	UAP/UGPA/PG M	43
215451	UDP-glucose 6-dehydrogenase	UGDH	44
271881	UDP-glucose 6-dehydrogenase	UGDH	45
209086	UDP-N-acetylglucosamine diphosphorylase/ UTP--glucose OR galactose-1-phosphate uridylyltransferase	UAP/UGPA/PG M (UAP1_1)	46
170890	fructose-1,6-bisphosphatase	FBP	47
201390	membrane-bound fructose-1,6-bisphosphatase	FBP (FBP1_2)	48
216851	glucokinase	GLK	49
158118	membrane-bound fructose-1,6-bisphosphatase	FBP (FBP1_1)	50
192661	glucokinase	GLK	51
255025	fructose-1,6-bisphosphatase	FBP (FBP2_2)	52
264529	galactokinase	GALK	53
191226	ABC transporter	ABC (ABC_1)	54
274700	chaperone ABC transporter	ABC	55
253792	glucosamine-phosphate N-acetyltransferase	GNA	56
178079	ABC transporter	ABC	57
256133	chitin synthase	CHS (CHS1_2)	58
160331	glucokinase	GLK	59
197697	chitin synthase	CHS (CHS1_1)	60
