# Supplementary material for: Identifying metabolic pathways for production of extracellular polymeric substances by the diatom Fragilariopsis cylindrus inhabiting sea ice
Source: ISME J. 2018 Jan 18;12(5):1237–51. doi: 10.1038/s41396-017-0039-z (PMC5932028; doi:10.1038/s41396-017-0039-z)
Supplement: Supplementary file 8 — Supplementary Table S3 [file 41396_2017_39_MOESM8_ESM.pdf]

Table S3 a-e: Top 10 genes and divergent alleles in *Fragilariopsis cylindrus* most highly up-regulated ($p \leq 0.001$) during phase II (**a**). III (**b**). IV (**c**). V (**d**) and VI (**e**) relative to open seawater phase I. Lists are ranked according to log2-fold changes (logFC) and Joint Genome Institute protein identifiers (protein ID) and associated annotations are provided.

Table S3 a: Phase II vs I

protein ID	logFC	annotation (Pfam/SignalP)
249397	8.59	HSF-type DNA-binding (PF00447)
250810	7.54	unknown with signal peptide (*)
255344	6.98	unknown with signal peptide (*)
246618	6.96	unknown with signal peptide
237465	6.27	Mycoplasma protein of unknown function. DUF285 (PF03382)
199400	5.94	Tetratricopeptide repeat (PF00515)
151943	5.78	PT repeat (PF04886)
254665	5.40	unknown
254692	5.38	unknown

Table S3 b: Phase III vs I

protein ID	logFC	annotation (Pfam/SignalP)
249397	9.83	HSF-type DNA-binding (PF00447)
209887	9.54	Chlorophyll A-B binding protein (PF00504); Lhcx3
246618	8.04	unknown with signal peptide
237465	7.86	Mycoplasma protein of unknown function. DUF285 (PF03382)
264517	7.75	short chain dehydrogenase (PF00106)
270990	7.34	unknown with signal peptide
151943	7.05	PT repeat (PF04886)
251935	6.83	unknown
189740	6.70	Fructose-bisphosphate aldolase class-I (PF00274); FBP_I

Table S3 c: Phase IV vs I

protein ID	logFC	annotation (Pfam/SignalP)
250810	7.20	unknown with signal peptide (*)
255344	6.90	unknown with signal peptide (*)
189740	6.85	Fructose-bisphosphate aldolase class-I (PF00274); FBP_I
154710	6.84	Ankyrin repeat (PF00023)
250890	6.18	Integrase core domain (PF00665)
242769	5.98	unknown
238597	5.70	unknown
164171	5.62	CbiX (PF01903)
261116	5.59	unknown

Table S3 d: Phase V vs I

protein ID	logFC	annotation (Pfam/SignalP)
189740	7.06	Fructose-bisphosphate aldolase class-I (PF00274); FBP_I
145453	6.91	PT repeat (PF04886)
154710	6.76	Ankyrin repeat (PF00023)
161472	6.70	antifreeze protein
235957	6.55	Exo_endo_phos (PF03372)
250987	6.53	unknown with signal peptide
191878	6.40	unknown
246775	6.40	MYND finger (PF01753)
250810	6.34	unknown with signal peptide

Table S3 e: Phase VI vs I

protein ID	logFC	annotation (Pfam/SignalP)
250810	7.66	unknown with signal peptide (*)
255344	7.28	unknown with signal peptide (*)
242769	7.25	unknown
189740	6.85	Fructose-bisphosphate aldolase class-I (PF00274); FBP_I
140452	6.46	unknown with signal peptide
154710	6.21	Ankyrin repeat (PF00023)
161472	5.88	antifreeze protein
164171	5.84	CbiX (PF01903)
237465	5.80	Mycoplasma protein of unknown function. DUF285 (PF03382)
